# Supplementing probiotics during intermittent fasting proves more effective in restoring ileum and colon tissues in aged rats

**DOI:** 10.1111/jcmm.18203

**Published:** 2024-03-06

**Authors:** Hikmet Taner Teker, Taha Ceylani, Seda Keskin, Gizem Samgane, Hüseyin Allahverdi, Eda Acikgoz, Rafig Gurbanov

**Affiliations:** ^1^ Department of Molecular Biology Ankara Medipol University Ankara Turkey; ^2^ Department of Molecular Biology and Genetics Muş Alparslan University Muş Turkey; ^3^ Department of Food Quality Control and Analysis Muş Alparslan University Muş Turkey; ^4^ Department of Histology and Embryology Van Yuzuncu Yil University Van Turkey; ^5^ Department Biotechnology, Institute of Graduate Education Bilecik Şeyh Edebali University Bilecik Turkey; ^6^ Department of Bioengineering Bilecik Şeyh Edebali University Bilecik Turkey; ^7^ Central Research Laboratory Bilecik Seyh Edebali University Bilecik Turkey

**Keywords:** aging, intermittent fasting, intestinal tissue, NF‐κB, SCD probiotics, TNF‐α

## Abstract

This study aimed to explore the impact of SCD Probiotics supplementation on biomolecule profiles and histopathology of ileum and colon tissues during a 30‐day intermittent fasting (IF) program. Male Sprague–Dawley rats, aged 24 months, underwent 18‐h daily fasting and received 3 mL (1 × 108 CFU) of SCD Probiotics. The differences in biomolecule profiles were determined using FTIR Spectroscopy and two machine learning techniques, Linear Discriminant Analysis (LDA) and Support Vector Machine (SVM), which showed significant differences with high accuracy rates. Spectrochemical bands indicating alterations in lipid, protein and nucleic acid profiles in both tissues. The most notable changes were observed in the group subjected to both IF and SCD Probiotics, particularly in the colon. Both interventions, individually and in combination, decreased protein carbonylation levels. SCD Probiotics exerted a more substantial impact on membrane dynamics than IF alone. Additionally, both IF and SCD Probiotics were found to have protective effects on intestinal structure and stability by reducing mast cell density and levels of TNF‐α and NF‐κB expression in ileum and colon tissues, thus potentially mitigating age‐related intestinal damage and inflammation. Furthermore, our results illustrated that while IF and SCD Probiotics individually instigate unique changes in ileum and colon tissues, their combined application yielded more substantial benefits. This study provides evidence for the synergistic potential of IF and SCD Probiotics in combating age‐related intestinal alterations.

## 1 INTRODUCTION

Contemporary aging and related disease treatment strategies aim to prolong lifespan, decelerate aging and enhance health span.^1^ Emerging evidence indicates intermittent fasting (IF) as a potent approach to these aims.^2^ IF denotes the periodic restriction of caloric intake for 16–24 h, employed for medicinal, social, or religious reasons.^3^ It offers protective benefits against chronic ailments, including type 2 diabetes, heart disease, neurodegenerative disorders, inflammatory bowel disease and multiple cancers.^4^ IF exceeding 16 h stimulates cellular‐level regeneration, reflecting in tissue and organ rejuvenation by activating autophagy mechanisms.^5^ Animal models demonstrate IF mitigates neuroinflammation, augments brain structure, and bolsters cognitive performance, aspects compromised by aging and Alzheimer's disease.^6^ Furthermore, it has been observed that ingestion of IF promotes a healthy balance of the gut microbiome.^7^

Dysbiosis, a disruption in the gut microbiota, significantly impacts aging processes.^8^, ^9^ The human gut microbiome, harbouring roughly 10^13^–10^14^ bacteria, facilitates critical host functions including food decomposition, lipid metabolism, vitamin synthesis, microbial control and intestinal barrier integrity preservation.^10^, ^11^, ^12^ Probiotic bacteria have exhibited efficacy in dysbiosis prevention and treatment.^13^, ^14^, ^15^ The Food and Agriculture Organization of the United Nations (FAO) and the World Health Organization (WHO) define probiotics as ‘live microorganisms that, when administered in adequate amounts, confer a health benefit on the host.’^16^ These include lipid profile enhancement, mycotoxin reduction, hypertension alleviation, blood glucose tolerance improvement and diabetes management.^17^ Probiotics also modulate the immune response by promoting mucin production and tight junction protein expression, thereby reducing colonic inflammation and enhancing intestinal barrier functionality.^18^

Infrared (IR) spectroscopy, a highly effective and versatile analytical technique utilized in the field of biology, employs its multi‐processing capabilities to rapidly and non‐invasively gather extensive data from biological samples. By capturing the vibrations of molecules in the mid‐IR region, it generates spectral bands that can be analysed to provide valuable information about the chemical composition and structure of the samples.^19^, ^20^ When combined with machine learning algorithms and other statistical data processing methods, IR spectroscopy has shown great promise in the diagnosis of a wide range of diseases.^21^, ^22^, ^23^ This technique has been used to analyse various biological samples such as blood, urine, and tissue, providing accurate and non‐invasive diagnosis of diseases such as cancer, diabetes, and cardiovascular disease.^24^, ^25^ Overall, IR spectroscopy is a powerful and promising technique that has the potential to revolutionize the field of medical diagnosis and treatment, offering non‐invasive, fast, and accurate analysis of biological samples.^26^

In this study, we aim to investigate the combined impact of IF and SCD Probiotics on the biomolecule profiles and histopathology of ileum and colon tissues in aged rats. We hypothesize that the co‐application of IF and SCD Probiotics could offer enhanced benefits in mitigating age‐related intestinal alterations, thereby presenting a promising approach for healthy aging.

## 2 MATERIAL METHODS

This section is presented in the Supporting Information.

## 3 RESULTS

### 3.1 The body weight, water and food consumption of animals

Significant weight loss was observed in the fasting groups, with less weight loss recorded in the FstPrb group. The lower significance value for weight loss between the Fst group and the Prb group compared to the control suggests the probiotic's role in weight gain stabilization. Despite the increased feed consumption by animals in the fasting groups due to adaptation, there was no significant difference in water consumption among the groups, as reported in our previous study.^27^

### 3.2 All groups exhibited differences in the lipid, protein and nucleic acid profiles of ileum tissue

**FIGURE 1:**
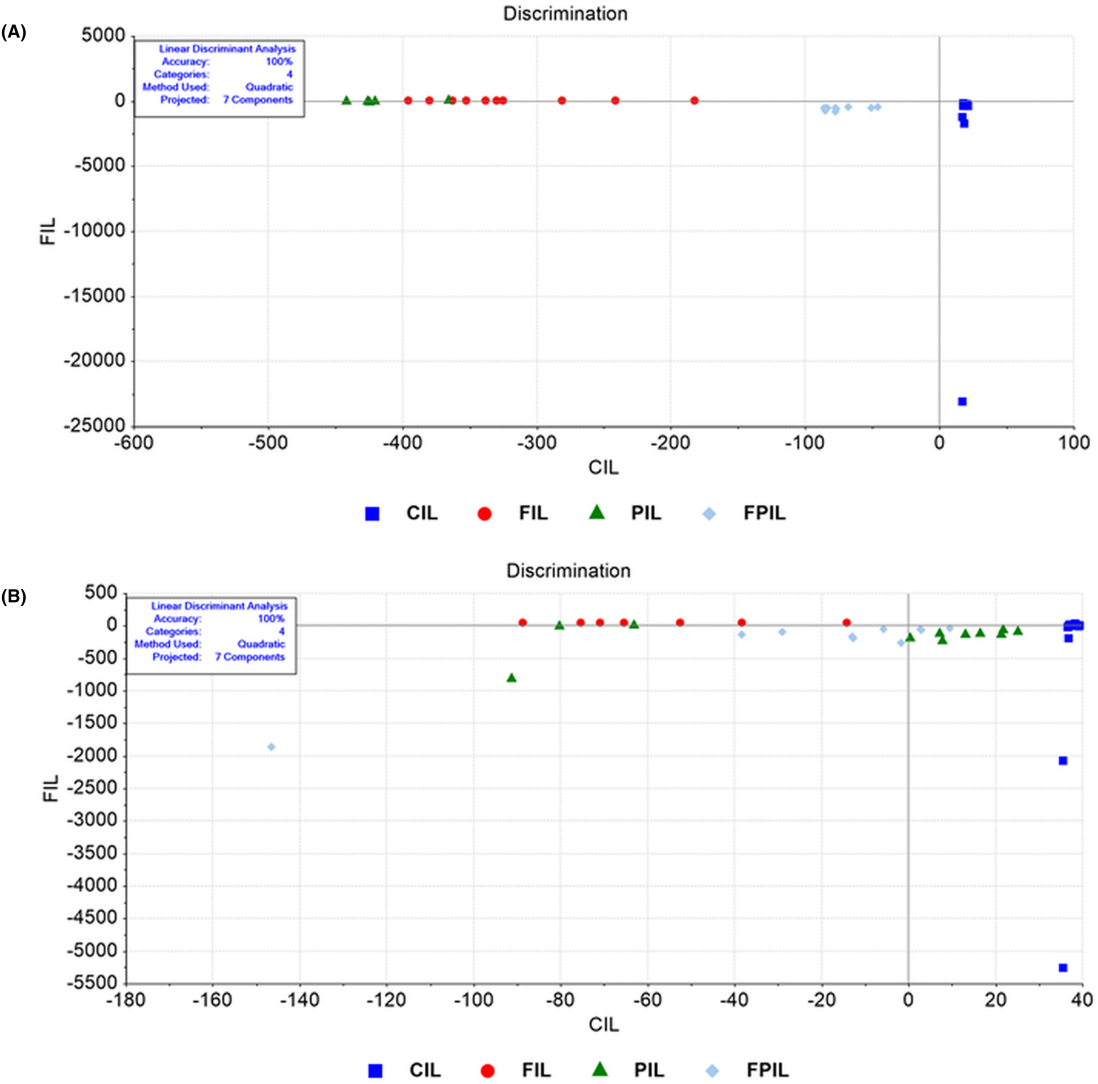
LDA discrimination plot for ileum samples in the full (4000–650 cm^−1^) spectral region (A). Control group and application groups significantly differed in overall biomolecule content, with 100% accuracy for whole content. LDA discrimination plot in the lipid (3000–2700 cm^−1^) spectral region (B). Control group and application groups clustered in distinct regions. Largest difference was seen in SCD Probiotics versus control, but similar to intermittent fasting effect. CIL (control), FIL (intermittent fasting), PIL (SCD Probiotics) and the FPIL applications (in which the intermittent fasting and SCD Probiotics were applied together).

The LDA method analysed the data, revealing significant differences in whole biomolecule content between the control group and groups receiving different applications (100% accuracy) (Figure 1A, Tables S1 and S2). The LDA plot showed distinct clusters for each group (Figure 1A), with the largest difference between SCD Probiotics (PIL) and control (CIL), similar to intermittent fasting (FIL). The group with both IF and SCD Probiotics (FPIL) was closer to the control. Lipid profiles also significantly differed (100% accuracy) (Figure 1B, Tables S3 and S4), with the largest separation in the intermittent fasting group. SCD Probiotics and combined use of IF and SCD Probiotics had similar effects but were closer to the control. Protein and nucleic acid content showed significant differentiation (100% accuracy) (Figures S1 and S2; Tables S5–S8), with combined use of IF and SCD Probiotics displaying different separation for proteins but similar patterns for nucleic acids, closer to the control. SVM classification demonstrated high training (97.5%) and validation accuracies (97.73%) for the ileum's biomolecular content (Figure S3).

The average spectra (4000–650 cm^−1^) for ileum samples showed distinct changes in various spectrochemical bands associated with specific biomolecule functional groups (Figure S4A–F). Key bands analysed were at 2955 cm^−1^ (CH3 antisymmetric stretching: Lipids and proteins), 2922 cm^−1^ (CH2 antisymmetric stretching: Lipids), 1740 cm^−1^ (C=O stretching: cholesterol ester), 1653 cm^−1^ (Amide I: α‐helical structure of proteins), 1545 cm^−1^ (Amide II: β‐sheet structure of proteins) and 1239 cm^−1^ (PO2 antisymmetric stretching: Nucleic acids). The CH3 antisymmetric value decreased significantly in the intermittent fasting group, with a notable increase in the group receiving both treatments (Figure S4A). The CH2 antisymmetric band increased in the fasting group, decreased in the SCD Probiotics group, and increased again in the combined treatment group (Figure S4B). The C=O stretching band decreased across all groups, most significantly in the fasting group (Figure S4C). Amide I and Amide II bands dropped in both fasting and SCD Probiotics groups, with no significant difference in the combined treatment group (Figure S4D,E). The PO2 antisymmetric band decreased in fasting and SCD Probiotics groups but increased in the combined treatment group (Figure S4F).

**FIGURE 2:**
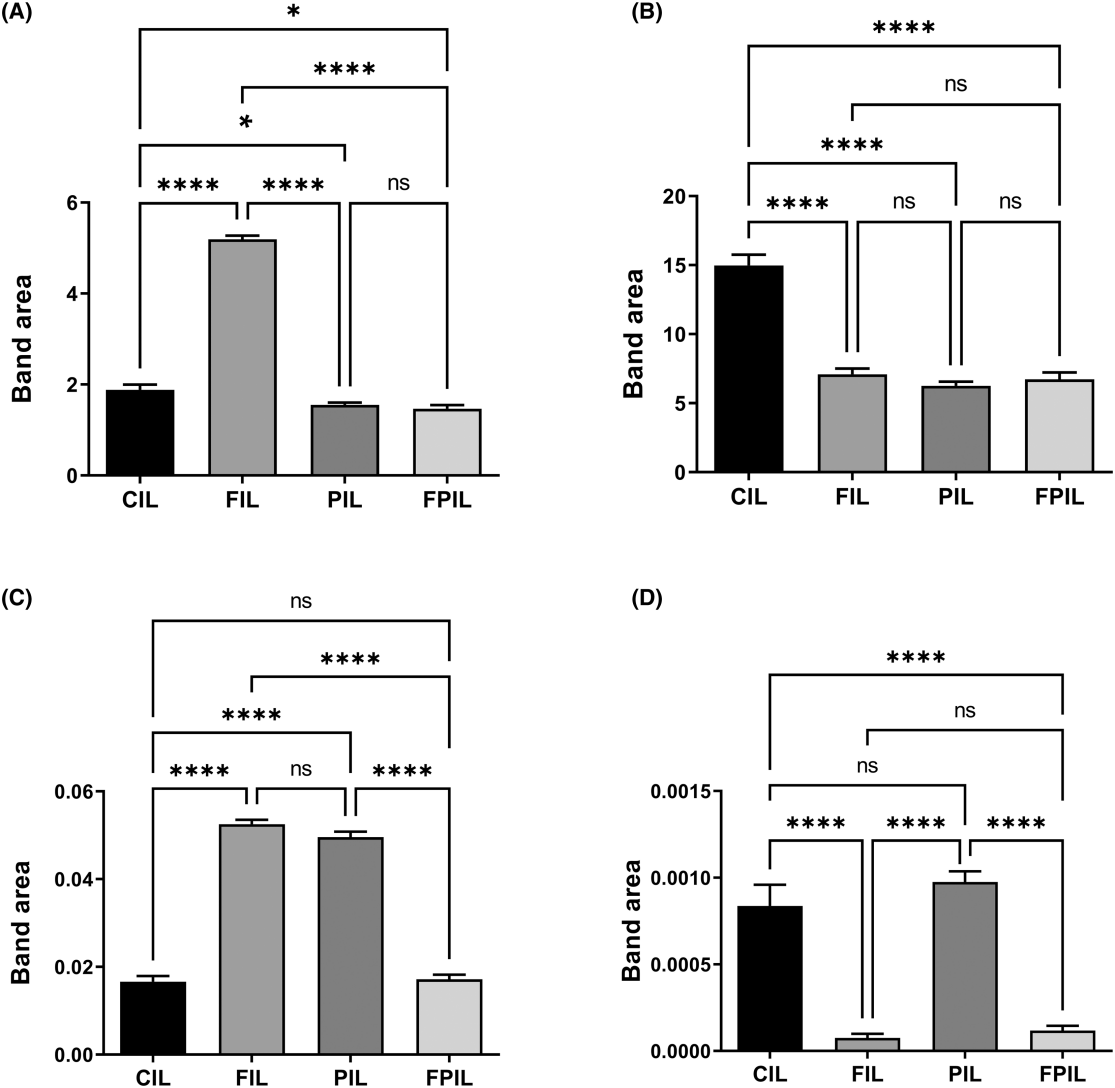
Quantitative changes in ileum‐associated spectrochemical parameters include band area ratios for: (A) Acyl chain length of fatty acids (A_2922_/A_2955_): IF group saw an increase; SCD Probiotics and combined treatment groups showed a decrease, (B) Protein phosphorylation (A_1239_/A_2955_): Decreased in all groups, (C) protein conformation (A_1653_/A_1545_) and (A_1080_/A1_545_): (A_1653_/A_1545_) increased in IF and SCD Probiotics, but unchanged in combined treatment; (A_1080_/A_1545_) increased in IF and SCD Probiotics but unchanged in combined treatment, (D) protein carbonylation (A_1740_/A_1545_): Decreased in IF and combined treatment; no change in SCD Probiotics group. The data were analysed using One‐way anova and unpaired *t*‐test, and the significance levels were stated as *p < 0.05 and ****p ≤ 0.0001. Decreased in IF and combined treatment; no change in SCD Probiotics group. CIL (control), FIL (intermittent fasting), PIL (SCD Probiotics) and the FPIL applications (in which the intermittent fasting and SCD Probiotics were applied together).

The areas of bands associated with acyl chain of fatty acids (A_2922_/A_2955_), protein phosphorylation (A_1239_/A_2955_), protein phosphorylation (A_1080_/A_1545_) and protein carbonylation (A_1740_/A_1545_) were significantly changed (Figure 2A–D). In the intermittent fasting group, the acyl chain of fatty acids increased significantly, while it decreased in both the SCD Probiotics group and the group receiving both treatments (Figure 2A). As for the protein phosphorylation (A_1239_/A_2955_) band, it decreased in all groups. However, the protein phosphorylation (A_1080_/A_1545_) increased in both the intermittent fasting and SCD Probiotics groups, but remained unchanged in the group receiving both treatments (Figure 2B,C). Protein carbonylation did not exhibit any change in the SCD Probiotics group. In contrast, it significantly decreased in both the intermittent fasting group and the group receiving both treatments (Figure 2D).

Significant changes were observed in bandwidths: CH_2_ antisymmetric (2922 cm^−1^: Lipids) decreased in all groups, most notably in the combined treatment group (Figure S5A), Amide I (1653 cm^−1^: α‐helical structure of proteins) decreased across all groups (Figure S5B). Membrane dynamics (A_2922_/A_2955_) showed a greater decrease in IF than SCD Probiotics, with the largest decrease in the combined treatment group (Figure S5C).

### 3.3 All groups exhibited differences in the lipid, protein and nucleic acid profiles of colon tissue

**FIGURE 3:**
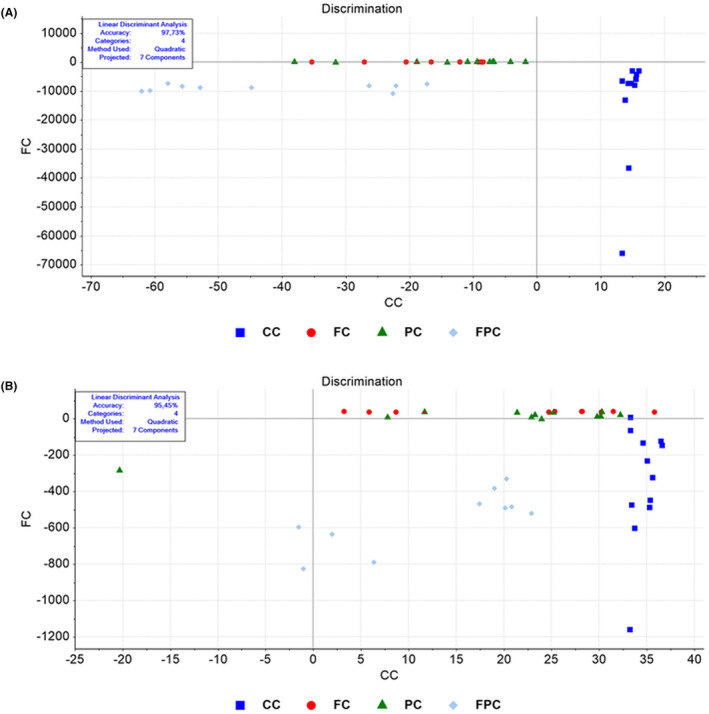
LDA discrimination plot for colon samples in the full (4000–650 cm^−1^) spectral region (A). LDA analysis revealed significant differentiation between control and application groups, with 97.73% accuracy rate. LDA discrimination plot for colon samples in the lipid (3000–2700 cm^−1^) spectral region (B). Lipid profiles showed significant differentiation with 95.45% accuracy. CC (control), FC (intermittent fasting), PC (SCD Probiotics) and the FPC applications (in which the intermittent fasting and SCD Probiotics were applied together).

The findings from the LDA analysis demonstrated a substantial divergence in the whole biomolecule content of colon tissue between the control group (CC) and the intermittent fasting (FC), SCD Probiotics (PC), and combined intermittent fasting and SCD Probiotics (FPC) groups. The analysis achieved a high level of accuracy, with 97.73% accuracy reported (Figure 3A; Tables S9 and S10). Intermittent fasting and SCD Probiotics groups had similar structures, while the combined treatment group showed the most distinct structure and separation. Lipid profiles also displayed significant differentiation (95.45% accuracy) (Figure 3B; Tables S11 and S12), with the most distinct separation in the combined treatment group. Protein and nucleic acid content showed significant differentiation with 93.18% and 97.73% accuracy rates, respectively (Figures S6 and S7; Tables S13–S16). While all treatments caused similar differences in protein profiles, the combined treatment group had the most distinct nucleic acid profile. SVM classification demonstrated high training (97.5%) and validation accuracies (92.5%) for the colon's biomolecular content (Figure S8).

IR spectroscopy of colon tissue samples showed visible changes in spectrochemical bands related to the specific biomolecule functional groups (Figure S9A–G). Analysed bands were at positions 2955 cm^−1^ (CH_3_ antisymmetric stretching: Lipids and proteins), 2955 cm^−1^ (CH_2_ antisymmetric stretching: Lipids), 1740 cm^−1^ (C=O stretching: Cholesterol ester), 1653 cm^−1^ (Amide I: α‐helical structure of proteins), 1545 cm^−1^ (Amide II: β‐sheet structure of proteins), 1239 cm^−1^ (PO2 antisymmetric stretching: Nucleic acids) and 1080 cm^−1^ (PO2 symmetric stretching: Nucleic acids and phospholipid). CH_3_ antisymmetric levels significantly decreased in the SCD Probiotics group (Figure S9A). CH_2_ antisymmetric band was unchanged in the fasting group, increased in the SCD Probiotics group and increased significantly in the combined treatment group (Figure S9B). C=O stretching significantly increased only in the combined treatment group (Figure S9C). Amide I levels decreased exclusively in the fasting group, while Amide II levels decreased in both fasting and SCD Probiotics groups (Figure S9D,E). PO2 antisymmetric and symmetric bands increased in the SCD Probiotics group and the combined treatment group (Figure S9F,G).

**FIGURE 4:**
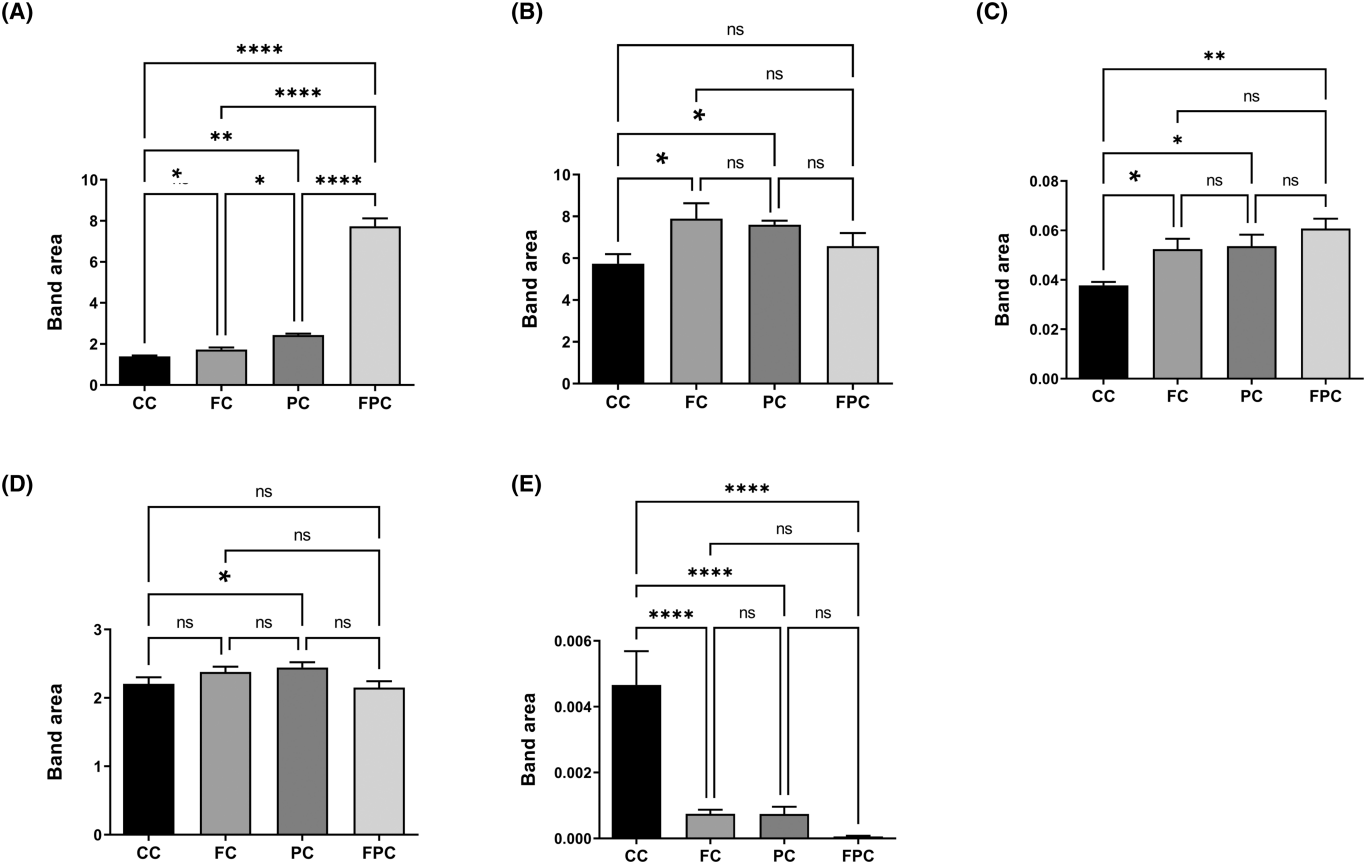
Quantitative changes in colon‐associated spectrochemical parameters include band area ratios for: (A) acyl chain length of fatty acids (A_2922/_A_2955_), (B) protein phosphorylation (A_1239_/A_2955_): Increased in IF and SCD Probiotics groups, (C) protein phosphorylation (A_1080_/A_1545_): Increased in all groups, (D) protein conformation (A_1653_/A_1545_): Increased only in SCD Probiotics group, (E) protein carbonylation (A_1740_/A_1545_): Decreased in all groups, with the greatest decrease in the combined treatment group. The data were analysed using one‐way anova and unpaired *t*‐test, and the significance levels were stated as *p < 0.05, **p ≤ 0.01 and ****p ≤ 0.0001. CC (control), FC (intermittent fasting), PC (SCD Probiotics) and the FPC applications (in which the intermittent fasting and SCD Probiotics were applied together).

The band areas of the acyl chain of fatty acids (A_2922_/A_2955_), protein phosphorylation (A_1239_/A_2955_), protein phosphorylation (A_1080_/A_1545_), protein conformation (A_1653_/A_1545_) and protein carbonylation (A_1740_/A_1545_) were significantly changed (Figure 4A–E). The acyl chain of fatty acids increased in all groups, with the most significant increase observed in the group receiving both treatments (Figure 4A). Protein phosphorylation (A_1239_/A_2955_) increased in both the intermittent fasting and SCD Probiotics groups, while protein phosphorylation (A_1080_/A_1545_) increased across all groups (Figure 4B,C). Protein conformation experienced an increase solely in the SCD Probiotics group and remained unchanged in the other groups (Figure 4D). Lastly, protein carbonylation levels decreased in all groups. However, the greatest decrease was in the group in which the two treatments were applied together (Figure 4E).

Significant alterations were observed in bandwidths: CH_3_ antisymmetric (2955 cm^−1^: Lipids and proteins) increased in the SCD Probiotics group (Figure S10A), CH_2_ antisymmetric (2922 cm^−1^: Lipids) increased in both SCD Probiotics and combined treatment groups (Figure S10B) and Amide I (1653 cm^−1^: α‐helical structure of proteins) decreased in the combined treatment group (Figure S10C). Membrane dynamics (A2922/A2955) increased in SCD Probiotics and combined treatment groups (Figure S10D) suggesting SCD Probiotics' effectiveness.

### 3.4 Histopathological assessments in the ileum and colon tissues

**FIGURE 5:**
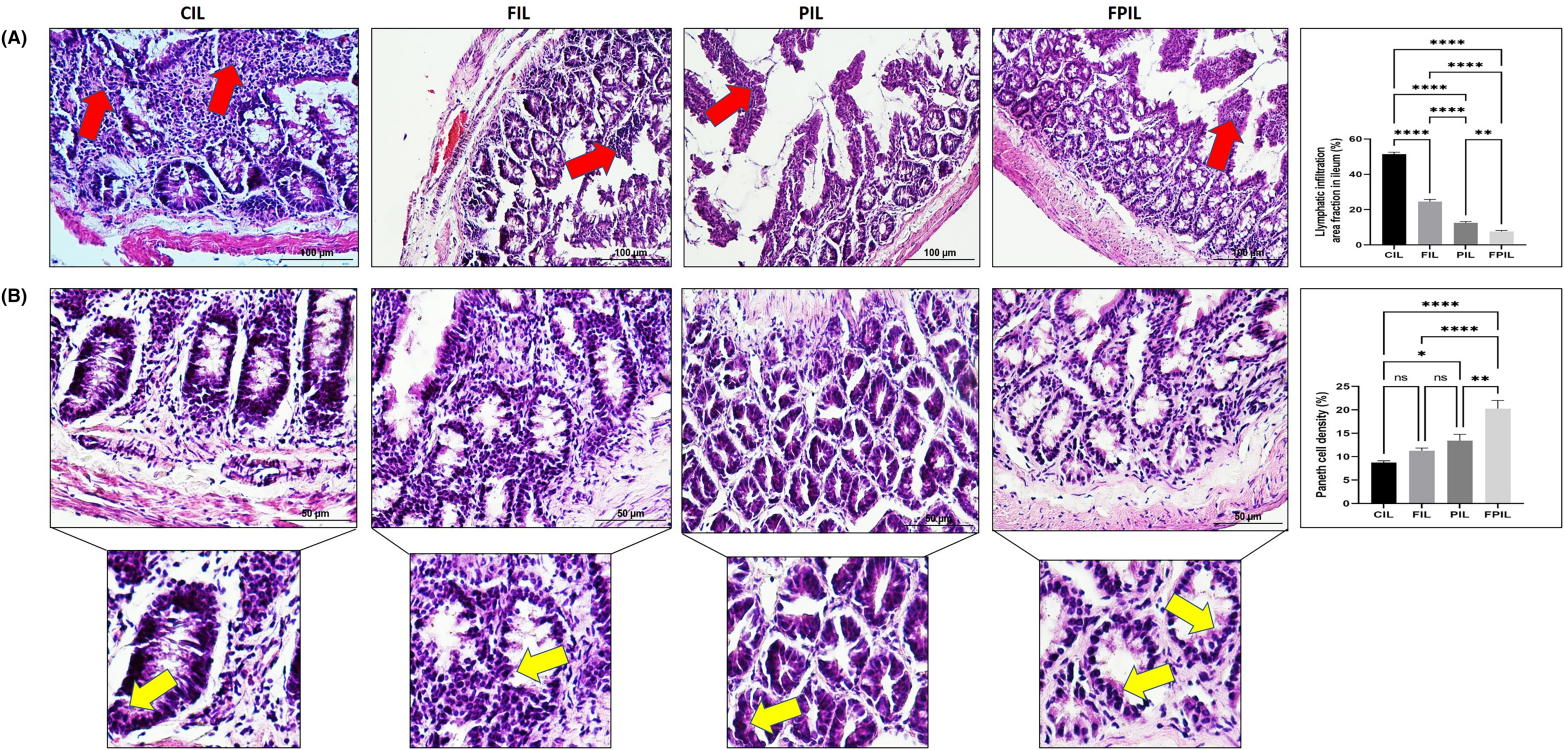
Representative images of H&E staining and quantification of lymphatic infiltration area fraction (%) in all groups of ileum tissues (A). Red arrow shows lymphatic infiltrates. Representative images of H&E staining and quantification of Paneth cells intensity of area fraction (%) in all groups of ileum tissues (B). Yellow arrow shows Paneth cells. Areas in the H&E‐stained microphotographs of all groups were magnified in the photos to which they belonged to the area of interest. Values are expressed as mean ± SEM; *n* = 7 rats in each group. The significance levels were stated as *p < 0.05, **p ≤ 0.01 and ****p ≤ 0.0001. (One‐way anova and nonparametric Mann–Whitney *U* test). Scale bar = 50 μm and 100 μm. CIL (control), FIL (intermittent fasting), PIL (SCD Probiotics) and the FPIL applications (in which the intermittent fasting and SCD Probiotics were applied together).

**FIGURE 6:**
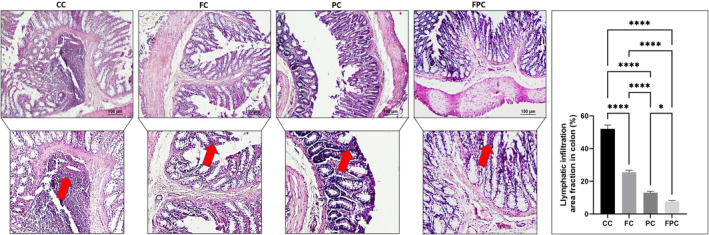
Representative images of H&E staining and quantification of lymphatic infiltration area fraction (%) in all groups of rat colon tissues. Red arrow shows lymphatic infiltrates. Areas in the H&E‐stained microphotographs of all groups were magnified in the photos to which they belonged to the area of interest. Values are expressed as mean ± SEM; *n* = 7 rats in each group. The significance levels were stated as *p < 0.05 and ****p ≤ 0.0001. (One‐way anova and nonparametric Mann–Whitney *U* test). Scale bar = 100 μm. CC (control), FC (intermittent fasting), PC (SCD Probiotics) and the FPC applications (in which the intermittent fasting and SCD Probiotics were applied together).

We investigated the effects of IF and SCD Probiotics on the ileum and colon histomorphology of aged rats and evaluated serial sections derived from each group. Through the histological inspection of both the ileum and colon H&E‐stained sections, a general morphological study was carried out. In the control group, there was shedding observed from the mucosal epithelial cells of both the ileum and colon. This resulted in the exposure of the lamina propria of the intestinal mucosa (as illustrated in Figures 5 and 6). In contrast, for IF and SCD Probiotics, the ileum and colon tissues exhibited a well‐defined structure. The mucosal epithelium and muscular layer remained intact, while the intestinal villi of the ileum were both copious and systematically arranged (shown in Figures 5 and 6). In addition, a more visible improvement in the ileum and colon histological architecture was detected in the group that received IF and SCD Probiotics compared to the group that only IF group. We observed that the mucosal structure was more regular, and the epithelial cells were more prominent. When a decrease in Paneth cell densities was observed in ileum samples of the control group, remarkably, there was a significant increase in the IF and SCD Probiotics recipient groups (seen Figure 5B). Our findings highlight the beneficial effects of IF and SCD Probiotics administration on aged gut health and structure, revealing potential areas of damage and mitigation in the aging process. Also, an increase in mucosal necrotic areas and glandular hyperplasia was detected with a decrease in goblet cell density due to increased lymphatic infiltration in the colon samples of control group (shown in Figure 6). On the contrary, it was remarkable that these pathologies were resolved in the groups receiving IF and SCD probiotics, but the curative effects of IF and SCD Probiotics together were more effective than only IF recipient group (see Figure 6).

### 3.5 IF and SCD Probiotics suppress age‐related gut inflammation

The one of the targets of our investigation was on the assessment of the inflammatory state in the aged ileum and colon tissues because inflammation is a hallmark of the aged rats. H&E‐stained sections from the ileum and colon of control rats exhibited enhanced lymphatic infiltration, indicative of inflammatory cells. Notably, the severity of this lymphatic infiltration was significantly attenuated in the IF and SCD Probiotics recipient group, when juxtaposed with the control and IF received group (Figures 5A and 6). The IF and SCD Probiotics appeared to exert a strong positive influence on inhibition of inflammation encountered within the ileum and colon mucosa. These findings thus underscore the potential of IF and SCD Probiotics treatments in mitigating the deleterious effects of aged ileum and colon on intestinal health.

### 3.6 Histomorphometric analysis of ileum and colon tissues

Aging is related with changes in the height, length, thickness, and depth of the ileum and colon tissues' histological structure over time. The collected morphometric data for the ileum and colon are depicted in Figures S11 and S12, respectively. Notably, both IF and SCD Probiotics groups showed significant differences in the lengths of the ileum and colon tissues.

To quantify intestinal measurements, we thoroughly examined histomorphometric data from the tissues of the ileum and colon, including the height of the villi, the depth of the crypts, the thickness of the mucosa and submucosa, and the total thickness of the intestinal wall. In the results obtained from this study, we found that villus lengths, crypt depths, mucosa, muscle layer and total wall lengths decreased, while the submucosa lengths not changed in the control rats (seen Figure S11). However, a significant increase in villus lengths, crypt depth, mucosal, muscle and total wall layers lengths was observed in the IF and SCD Probiotics given groups compared to the control group (seen Figure S11). According to the findings, IF and SCD Probiotics considerably improved every histomorphometric parameter that was examined in the aged ileum. The morphometric parameters analysis of the colon tissues of all groups is shown in Figure S12. We measured the crypt depth, mucosa, submucosa, muscle layer and total wall thickness parameters of the colon tissues. Our results displayed an increase in submucosa and muscle layer along with a decrease in crypt depths and mucosal lengths in the control group. Conversely, in the IF and SCD Probiotics given groups (shown in Figure S12), there was a significant decrease in submucosa and muscle layers, along with a significant increase in crypt depths and mucosal lengths. Although IF was not as much as SCD probiotics, it was observed that the colons of rats in the control group exhibited morphometric changes similar to the group given IF+ SCD Probiotics given group.

### 3.7 Measurements of mast cells in the ileum and colon

In this study, we investigated the role of mast cells because they have been associated with age‐related intestinal damage. As mast cells exhibit metachromasia, slices of the ileum and colon were stained with toluidine blue. Thus, violet‐purple mast cell densities in ileum and colon sections were examined. In the sections of the control group, the density of mast cells was predominantly detected in the intestinal serosa layers. In contrast, the mast cell density was significantly decreased in the IF and SCD Probiotics recipient groups. In the IF and SCD Probiotics groups, intestinal mast cells were readily distinguishable with the granules within these cells typically appearing homogenous. The density of serosal mast cells (in both ileum and colon) was significantly higher and showed a heightened presence of granules in the control group compared to IF and SCD Probiotics groups, as depicted in Figures S13 and S14.Thus, we demonstrated a strong link between the number of mast cells and intestinal inflammation in aged rats. These results highlight potential effects of IF and SCD Probiotics administrations to reduce mast cell proliferation and, in turn, intestinal inflammation.

### 3.8 IF and SCD Probiotics ameliorates intestinal inflammation via TNF‐α and NF‐κB expressions in the ileum and colon

**FIGURE 7:**
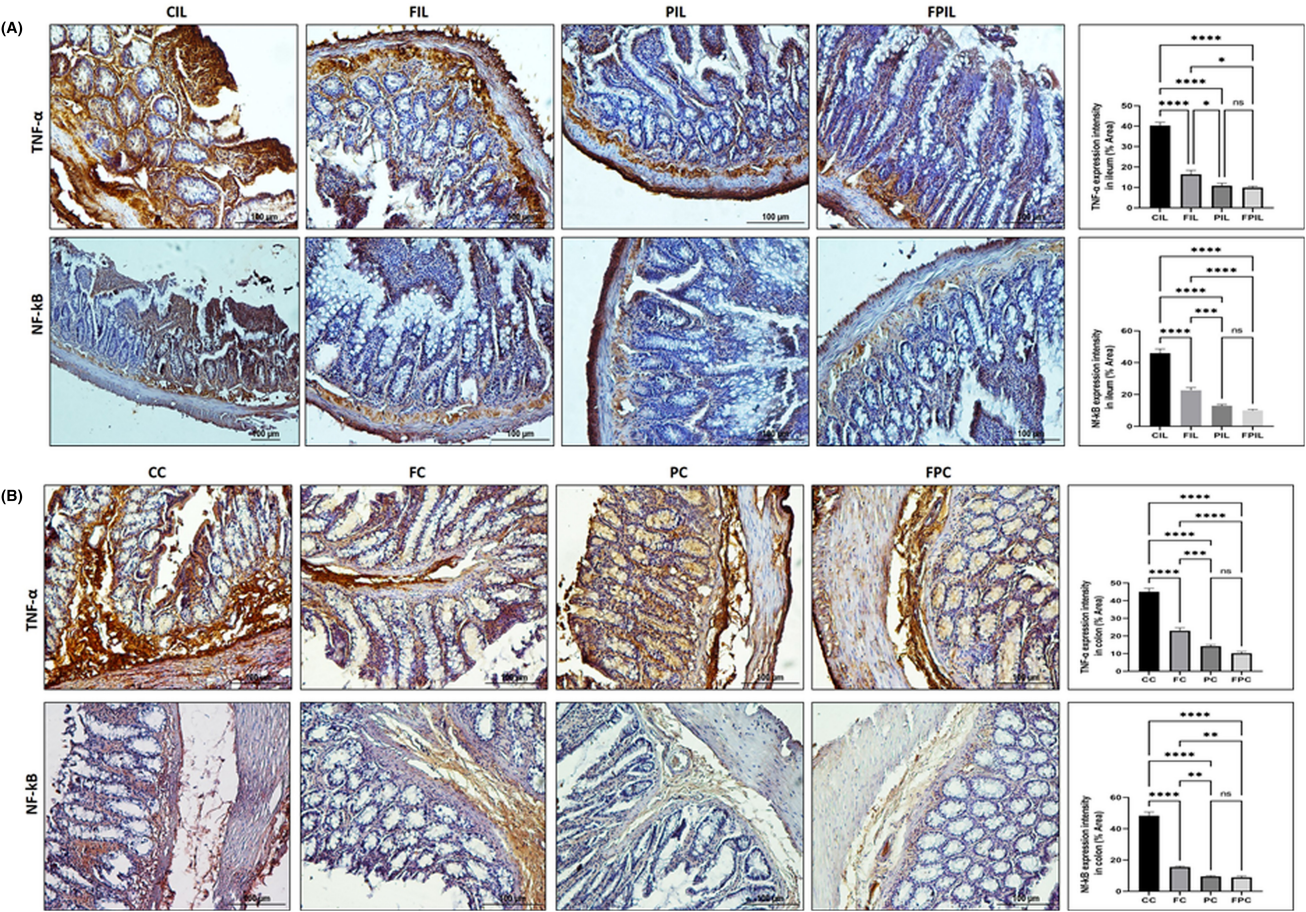
A TNF‐α and NF‐κB staining intensity in ileum (A) and colon (B). TNF‐α: Tumour Necrosis Factor‐alpha, NF‐κB: Nuclear factor kappa‐light‐chain‐enhancer of activated B cells. IHC staining images of TNF‐α and NF‐κB expression in the rat ileum. Graphs of TNF‐α and NF‐κB staining intensity in the ileum and colon tissues as measured in ImageJ (FIJI). Values are expressed as mean ± SEM; *n* = 7 rats in each group. The significance levels were stated as *p < 0.05, **p ≤ 0.01 ***p ≤ 0.001 and ****p ≤ 0.0001. (One‐way anova and nonparametric Mann–Whitney *U* test). (IHC staining, Scale bar: 100 μm). CIL (control), FIL (intermittent fasting), PIL (SDC Probiotics) and the FPIL applications (in which the intermittent fasting and SCD Probiotics were applied together); CC (control), FC (intermittent fasting), PC (SCD Probiotics), and the FPC applications (in which the intermittent fasting and SCD Probiotics were applied together).

We examined the effects of IF and SCD Probiotics on the production of the inflammatory cytokines TNF‐α and NF‐κB, which are frequently increased in the ileum and colon tissues of aged rats. The immunochemical analysis of TNF‐α and NF‐κB expression in the ileum and colon of control and other treatment groups are presented in Figure 7A,B. When the results were evaluated, it was seen that IF and SCD Probiotics treatments revealed less expressions of TNF‐α and NF‐κB compared to control group. TNF‐α expressions in the ileum and colon of the control group were significantly higher compared to the groups receiving IF and SCD probiotics. DAB‐positive stained TNF‐α cells were predominantly seen in the brown‐yellow coloured mucosa (lamina propria) and submucosa. The IF and SCD probiotics resulted in a significant reduction in TNF‐α levels for expressions in ileum and colon (****p ≤ 0.0001, seen Figure 7A,B). However, the ameliorative effects of IF and SCD probiotic treatment on NF‐κB expression were detected with significant decrease in ileum and colon tissues in the group receiving IF and SCD probiotics. DAB‐positive staining NF‐kB cells were predominantly seen in the brown‐yellow coloured mucosa (lamina propria) and submucosa. Figure 7A,B showed that the immunostaining intensity of NF‐κB increased in the control group compared to the other groups and decreased in the groups that received IF and SCD Probiotics(****p ≤ 0.0001).

## 4 DISCUSSION

Aging‐induced changes in the gut, characterized by tissue degradation and dysbiosis, have critical implications for overall health and well‐being. Key strategies, namely IF and probiotic supplementation, have been identified as potential interventions to counter these detrimental effects.^4^, ^26^, ^28^ This study probed the combined efficacy of IF and SCD Probiotics supplementation on the biomolecular composition and histological structure of ileum and colon tissues in aged rats. Through spectrochemical analysis and machine learning techniques, we observed distinct alterations in the lipid, protein, and nucleic acid profiles within the ileum and colon tissues. Importantly, the combination of IF and SCD Probiotics appeared to exert a more pronounced impact, particularly within the colon. The research underscored the complexity of age‐related intestinal health dynamics. For instance, as individuals age, the balance between microbiota and systemic host immunity systems experiences significant changes due to inflammation, affecting the integrity of the intestinal barrier.^29^ Our findings highlighted the protective effects of IF and SCD Probiotics treatments on the aged rat ileum and colon histology, potentially signalling their positive roles in mitigating age‐related immune dysregulation and suggest that the combined application of IF and SCD Probiotics could potentially offer more comprehensive benefits for gut health in aging. However, these results need to be interpreted in light of the complex interplay between the host's systemic immunity and gut microbiota, particularly as inflammation and age‐related shifts occur.

The intestinal epithelial tissue undergoes continuous renewal, a process that is hindered by aging, compromising the protective role of the epithelium.^30^ Aging‐induced alterations in the ileum and colon tissues of rats, as observed in our study, manifested as increased epithelial shedding, decreased density of goblet and Paneth cells, heightened inflammation, and morphometric changes. These perturbations potentially disrupt intestinal function, necessitating interventions to foster regeneration.^18^, ^24^, ^31^ Our findings demonstrate that both IF and SCD Probiotics can alleviate these adverse changes associated with aging, indicating their potential as therapeutic strategies. The intestinal epithelium's primary food source is short‐chain fatty acids (SCFAs), produced by bacterial fermentation of complex carbohydrates in the gut.^32^, ^33^, ^34^ IF enhances the production of these SCFAs, whereas SCD Probiotics, through constituents like *Bifidobacterium bifidum* and *Bacillus subtilis*, can increase their concentration by regulating other gut bacteria. The gut microbiota primarily comprises Firmicutes and Bacteroidetes, which produce important SCFAs, including butyrate, acetate and propionate. An elevated Firmicutes to Bacteroidetes (F/B) ratio is linked to numerous diseases and is known to increase with aging. Recently, our team found that both IF and SCD Probiotics play a crucial role in balancing this ratio and enhancing alpha diversity indices in the gut microbiota.^7^, ^15^ Moreover, IF and certain SCD Probiotics species, such as *Lactobacillus plantarum*, are known to stimulate autophagy mechanisms. These mechanisms remove misfolded or aggregated proteins and clear damaged organelles like mitochondria, endoplasmic reticulum, and peroxisomes, promoting cell regeneration in the intestinal epithelium.^4^, ^35^ These findings suggest a broader, more encompassing role for IF and SCD Probiotics in age‐related intestinal health management.

Aging is frequently correlated with escalated lipid peroxidation, culminating in oxidative damage.^14^, ^36^ This heightened oxidative stress is implicated in intensifying inflammation and fostering protein modifications, subsequently leading to the generation of reactive oxygen species and promoting lymphatic infiltration, a phenomenon that aligns with our observed results.^8^, ^37^ Our findings reveal that both IF and SCD Probiotics effectively mitigated lymphatic infiltration in aged rats, indicating their inherent anti‐inflammatory attributes.^38^, ^39^ Moreover, our study detected an increase in mast cell densities and up regulated TNF‐α and COX‐2 expression in aged rats, elements that contribute to oxidative stress and inflammation. Remarkably, both IF and SCD Probiotics counteracted these deleterious effects, suggesting their therapeutic potential against age‐related inflammatory conditions. In addition, protein carbonylation, an irreversible oxidative protein modification, is recognized as a pivotal indicator of oxidative stress‐associated disorders.^40^ Prior research posits that IF can attenuate protein carbonylation,^41^ a finding corroborated by our spectrochemical data for the ileum. Interestingly, while the sole application of SDC Probiotics did not significantly alter protein carbonylation, the concomitant application of IF and SCD Probiotics induced a marked decrease. These results underscore the potential benefit of synergistically applying IF and SCD Probiotics for managing oxidative stress‐associated changes in the aging gut. The combined application of IF and SCD Probiotics also had a greater effect on fatty acyl chain length, which plays crucial roles in forming unique interactions between individual lipid molecules and proteins and, at high concentrations, in determining the overall properties of membranes.^42^

## 5 CONCLUSION

This study demonstrates that the combined application of IF and SCD Probiotics induces unique and potentially beneficial changes in the ileum and colon tissues of aged rats. The notable alterations in lipid, protein and nucleic acid profiles suggest a restorative effect on the intestinal tissues. Furthermore, the marked decrease in protein carbonylation and inflammation indices (TNF‐α and NF‐κB) underlines the potential of this combined approach in mitigating age‐related intestinal damage and inflammation. The findings underscore the promise of utilizing a combined IF and SCD Probiotics regimen as part of a therapeutic strategy for healthier aging. Future studies should validate these findings and further explore the underlying mechanisms to pave the way for potential clinical applications.

## AUTHOR CONTRIBUTIONS

**Hikmet Taner Teker:** Conceptualization (equal); data curation (equal); formal analysis (equal); funding acquisition (equal); investigation (equal); methodology (equal); resources (equal); software (equal); supervision (equal); validation (equal); visualization (equal); writing – original draft (equal); writing – review and editing (equal). **Taha Ceylani:** Conceptualization (equal); data curation (equal); formal analysis (equal); funding acquisition (equal); investigation (equal); methodology (equal); resources (equal); software (equal); supervision (equal); validation (equal); visualization (equal); writing – original draft (equal); writing – review and editing (equal). **Seda Keskin:** Data curation (equal); formal analysis (equal); funding acquisition (equal); investigation (equal); methodology (equal); resources (equal); software (equal); supervision (equal); validation (equal); visualization (equal); writing – original draft (equal). **Gizem Samgane:** Investigation (equal); methodology (equal); resources (equal); software (equal); supervision (equal); validation (equal); visualization (equal). **Hüseyin Allahverdi:** Methodology (equal); supervision (equal); validation (equal); visualization (equal). **Eda Acikgoz:** Data curation (equal); formal analysis (equal); funding acquisition (equal); investigation (equal); methodology (equal); resources (equal); software (equal); supervision (equal); validation (equal); visualization (equal); writing – original draft (equal). **Rafig Gurbanov:** Data curation (equal); formal analysis (equal); funding acquisition (equal); investigation (equal); methodology (equal); resources (equal); software (equal); supervision (equal); validation (equal); visualization (equal); writing – original draft (equal); writing – review and editing (equal).

## FUNDING INFORMATION

This research received no specific grant from any funding agency in the public, commercial, or not‐for‐profit sectors.

## CONFLICT OF INTEREST STATEMENT

The authors have declared that no competing interests exist.

## Supporting information


Figure S1.

Table S1.

## Data Availability

The datasets generated during and/or analysed during the current study are available from the corresponding author on reasonable request.
