# Supplementary material for: Supplementing probiotics during intermittent fasting proves more effective in restoring ileum and colon tissues in aged rats
Source: J Cell Mol Med. 2024 Mar 6;28(6):e18203. doi: 10.1111/jcmm.18203 (PMC10915827; doi:10.1111/jcmm.18203)
Supplement: Supplementary file 1 — Figure S1. [file JCMM-28-e18203-s001.docx]

**Supplementing probiotics during intermittent fasting proves more effective in restoring ileum and colon tissues in aged rats**

Hikmet Taner Teker^1^, Taha Ceylani^2,3*^, Seda Keskin^4^, Gizem Samgane^5^, Hüseyin Allahverdi^2^, Eda Acikgoz^4^, Rafig Gurbanov^6,7*^

^1^Department of Molecular Biology, Ankara Medipol University Ankara, Turkey

^2^Department of Molecular Biology and Genetics, Muş Alparslan University Muş, Turkey

^3^Department of Food Quality Control and Analysis, Muş Alparslan University Muş, Turkey

^4^Department of Histology and Embryology, Van Yuzuncu Yil University, Van, Turkey

^5^Institute of Graduate Education, Department Biotechnology, Bilecik Şeyh Edebali University Bilecik, Turkey

^6^Department of Bioengineering, Bilecik Şeyh Edebali University Bilecik, Turkey

^7^Central Research Laboratory, Bilecik Seyh Edebali University Bilecik, Turkey

* Correspondence: [t.ceylani@alparslan.edu.tr](mailto:t.ceylani@alparslan.edu.tr) ORCID ID: 0000-0002-3041-6010 (T.Ceylani), [rafik.kurbanov@gmail.com](mailto:rafik.kurbanov@gmail.com) ORCID ID: 0000-0002-5293-6447 (R. Gurbanov)

**SUPPLEMENTARY FIGURES**

**Fig. S1.** LDA discrimination plot for ileum samples in protein (1700-1500 cm^-1^) spectral region. CIL (control) and FIL (intermittent fasting), PIL (SCD Probiotics) and FPIL (in which the IF and SCD Probiotics)

**Fig. S2.** LDA discrimination plot for ileum samples in spectral region in nucleic acids and polysaccharides (1200-650 cm^-1^). CIL (control), FIL (intermittent fasting), PIL (SCD Probiotics) and FPIL (in which the IF and SCD Probiotics were applied together)

**
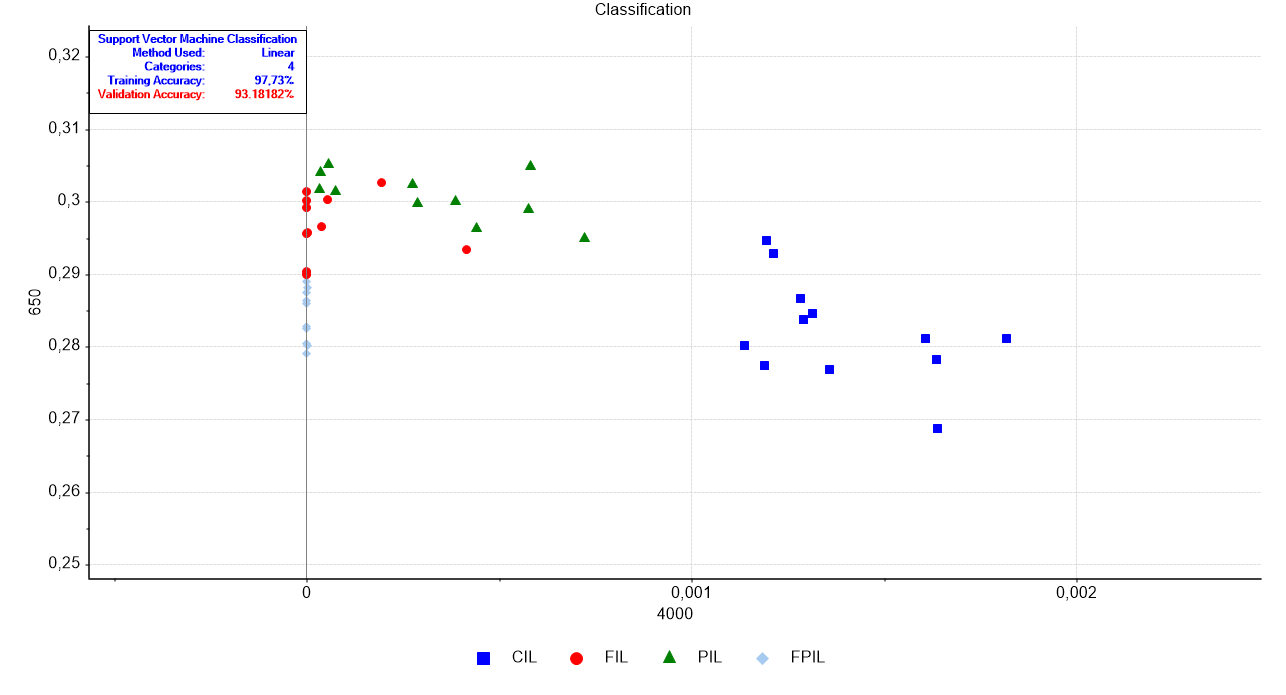
**

**Figure S3.** SVM classification plot for ileum samples in the full (4000-650 cm^-1^) spectral region. CIL (control), FIL (intermittent fasting), PIL (SCD Probiotics) and FPIL (in which the IF and SCD Probiotics were applied together)

**
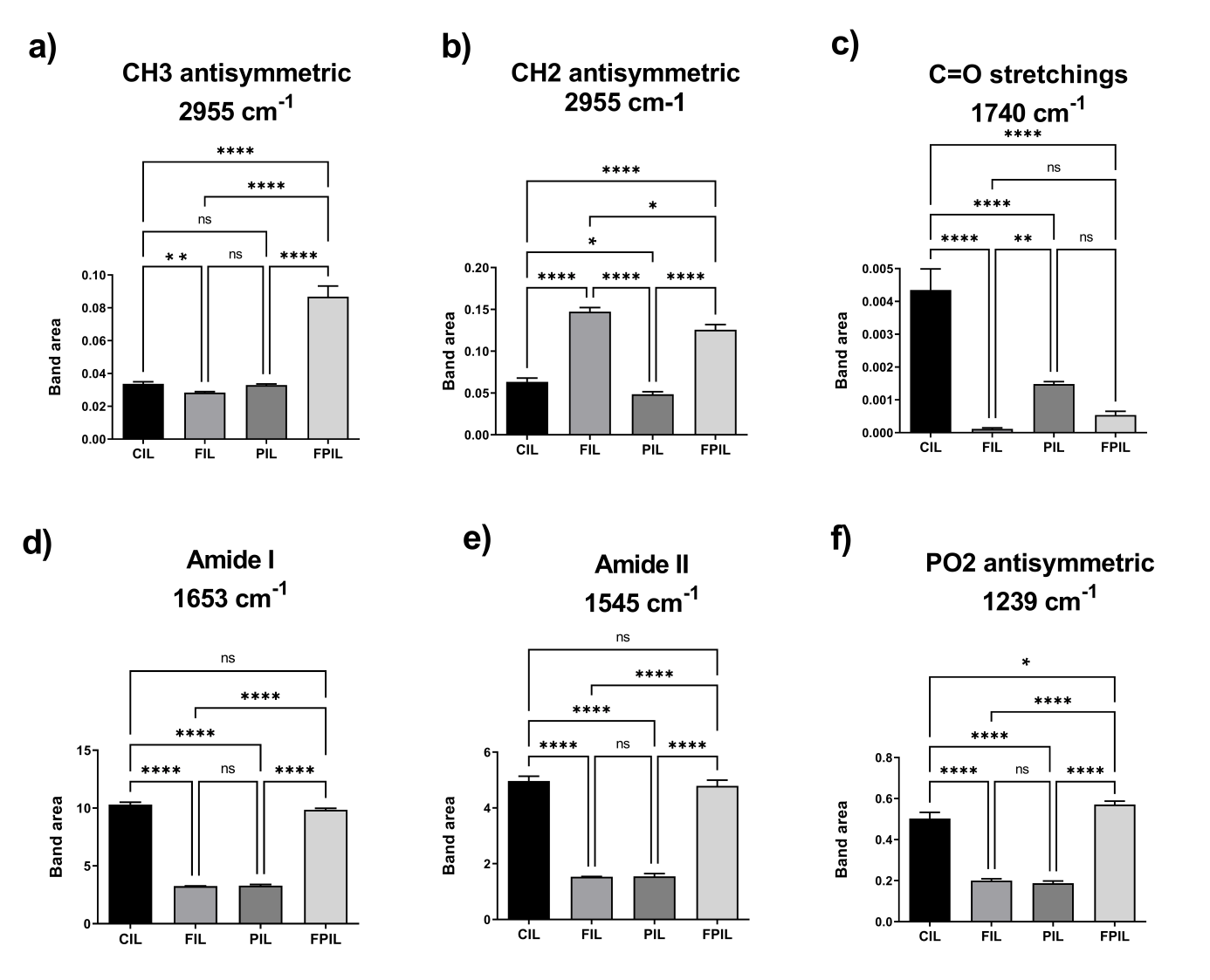
**

**Fig. S4.** Quantitative changes in ileum-associated spectrochemical parameters include: **a)** CH3 antisymmetric (2955 cm-1): IF group showed decreased value; combined treatment saw an increase, **b)** CH2 antisymmetric (2922 cm-1): IF group showed an increase; SCD Probiotics group decreased; combined treatment increased, **c)** Lipid carbonyl (C=O stretching, 1740 cm-1): All groups showed decreased values, most significantly in IF group, **d)** Amide I (1653 cm-1) and e) Amide II (1545 cm-1): Both bands dropped in IF and SCD Probiotics groups; no major change in combined treatment, **f)** PO2 antisymmetric (1239 cm-1): Band decreased in IF and SCD Probiotics groups; increased in combined treatment. The data were analyzed using One-way anova and unpaired t-test, and the significance levels were stated as P< 0.05 *, P ≤ 0.01 **, *, and P ≤ 0.0001 ****. CIL (control), FIL (Intermittent fasting)**,** PIL (SCD Probiotics), and the FPIL applications (in which the Intermittent fasting and SCD Probiotics were applied together).


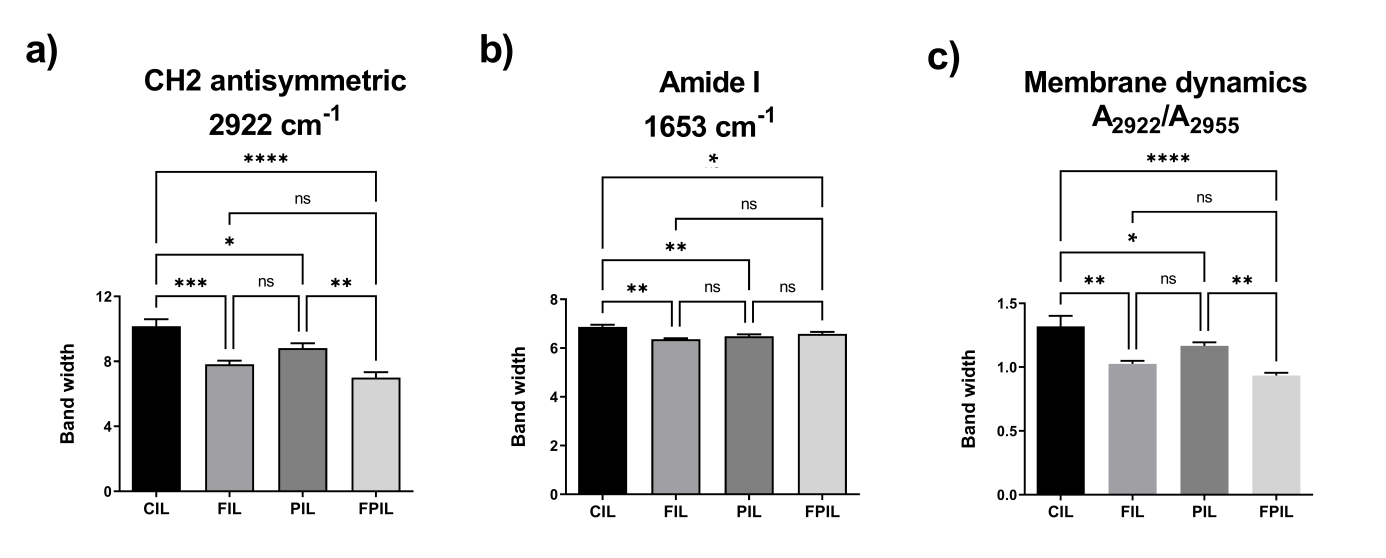


**Fig. S5.** The quantitative changes in band width spectrochemical parameters. The indices for **a)** 2922 cm^-1^ (CH_2_ antisymmetric stretching: lipids), **b)** 1653 cm^-1^ (Amide I: *α-*helical structure of proteins), and **c)** Membrane dynamics (A_2922_/A_2955_), CIL (control), FIL (intermittent fasting), PIL (SCD Probiotics) and FPIL (in which the IF and SCD Probiotics were applied together). The data were analyzed using One-way anova and unpaired t-test, and the significance levels were stated as P< 0.05*, P ≤ 0.01 **, P ≤ 0.001 ***, and P ≤ 0.0001 ****.

**Fig. S6.** LDA discrimination plot for colon samples in protein (1700-1500 cm^-1^) spectral region. CC (control) and FC (intermittent fasting), PC (SCD Probiotics) and the group FPC (in which the IF and SCD Probiotics were applied together)

**Fig. S7.** LDA discrimination plot for colon samples in spectral region in nucleic acids and polysaccharides (1200-650 cm-1). CC (control) and FC (intermittent fasting), PC (SCD Probiotics) and the group FPC (in which the IF and SCD Probiotics were applied together)

**
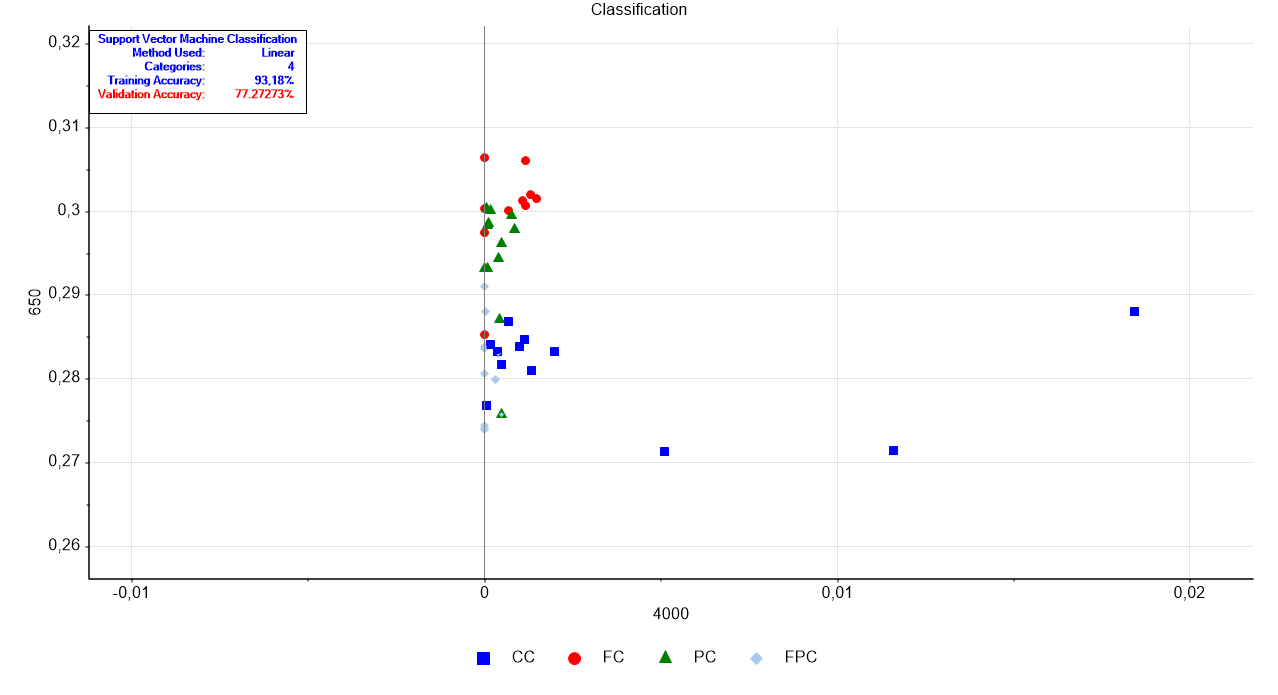
Figure S8.** SVM classification plot for ileum samples in the full (4000-650 cm^-1^) spectral region. CC (control) and FC (intermittent fasting), PC (SCD Probiotics) and the group FPC (in which the IF and SCD Probiotics were applied together)

**
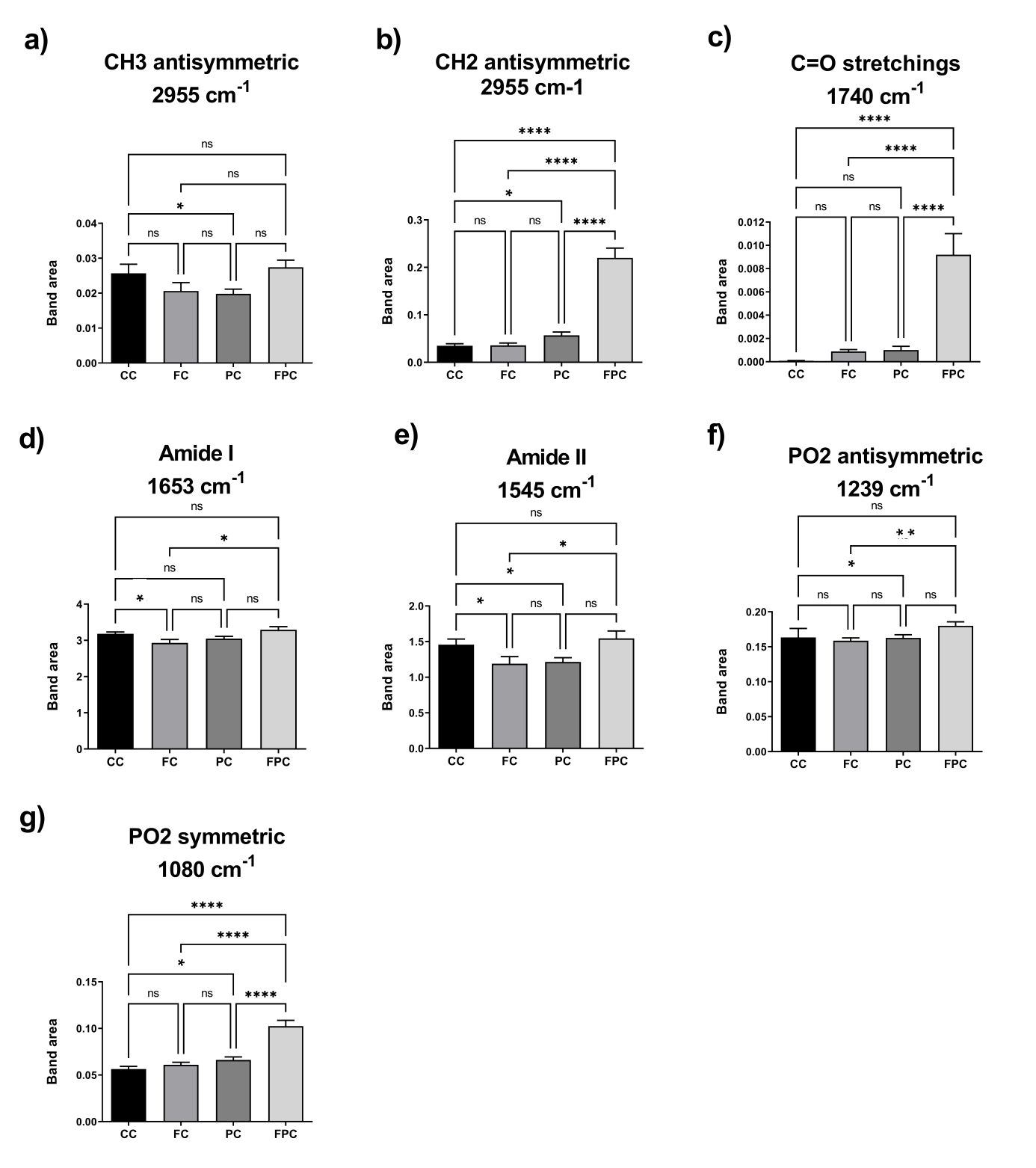
**

**Figure S9.** Quantitative changes in colon-associated spectrochemical parameters include band areas for: **a)** CH3 antisymmetric (2955 cm-1): SCD Probiotics group saw decreased levels, **b)** CH2 antisymmetric (2922 cm-1): Increased in SCD Probiotics and combined treatment groups, **c)** Lipid carbonyl (C=O stretchings, 1740 cm-1): Increased only in combined treatment group **d)** Amide I (1653 cm-1): Decreased in IF group, **e)** Amide II (1545 cm-1): Decreased in IF and SCD Probiotics groups, **f)** PO2 antisymmetric (1239 cm-1) and g) PO2 symmetric (1080 cm-1): Both increased in SCD Probiotics and combined treatment groups. The data were analyzed using One-way anova and unpaired t-test, and the significance levels were stated as P< 0.05 *, P ≤ 0.01 **, and P ≤ 0.0001 ****.CC (control), FC (Intermittent fasting)**,** PC (SCD Probiotics), and the FPC applications (in which the Intermittent fasting and SCD Probiotics were applied together).


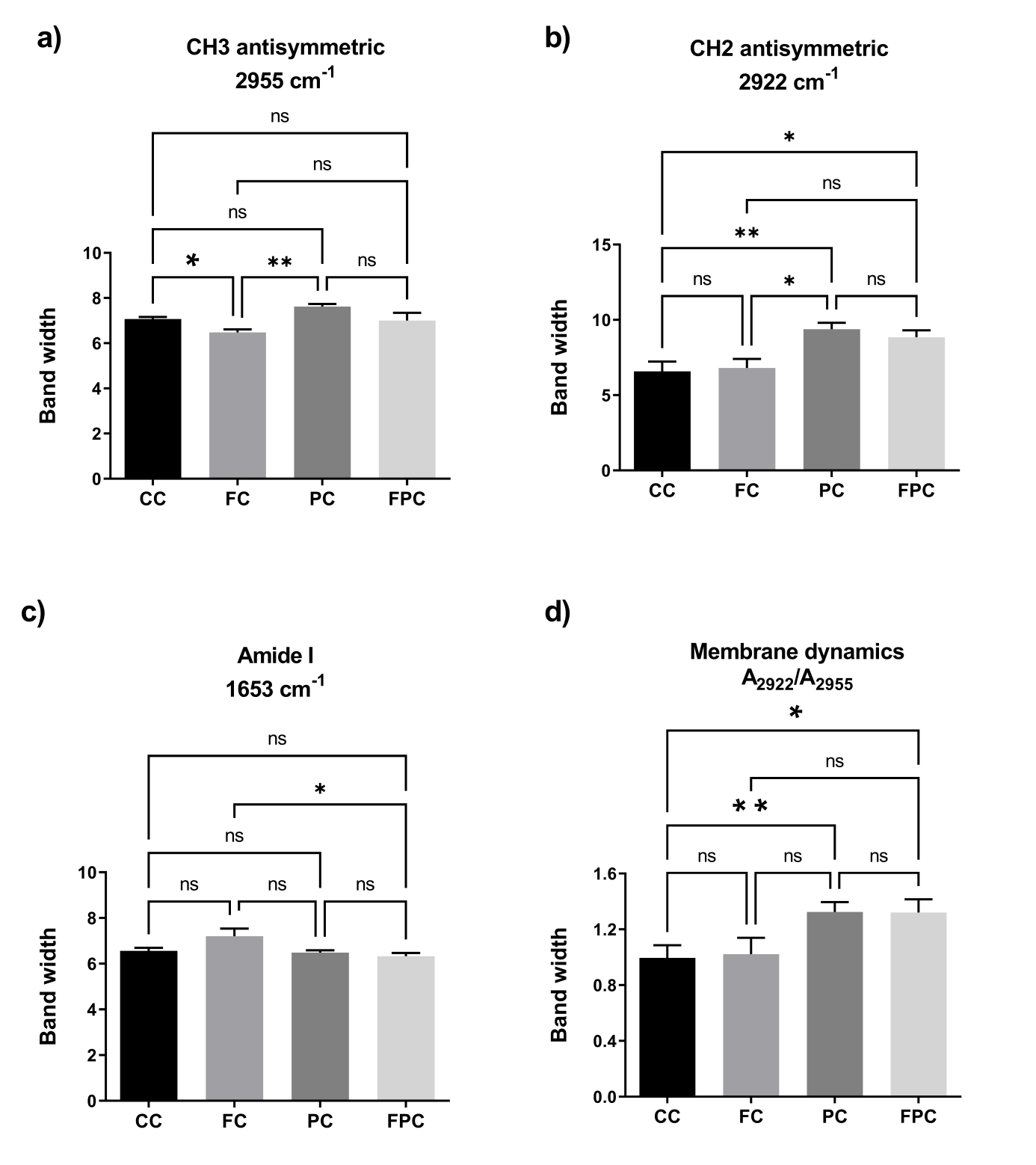


**Fig. S10.** The quantitative changes in band width spectrochemical parameters. The indices for **a)** 2955 cm^-1^ (CH_3_ antisymmetric stretching: lipids and proteins), **b)** 2922 cm^-1^ (CH_2_ antisymmetric stretching: lipids), **c)** 1653 cm^-1^ (Amide I: *α-*helical structure of proteins), and **d)** Membrane dynamics (A_2922_/A_2955_), CC (control) and FC (intermittent fasting), PC (SCD Probiotics) and the group FPC (in which the IF and SCD Probiotics were applied together). The data were analyzed using One-way anova and unpaired t-test, and the significance levels were stated as P< 0.05 * and P ≤ 0.01 ** .


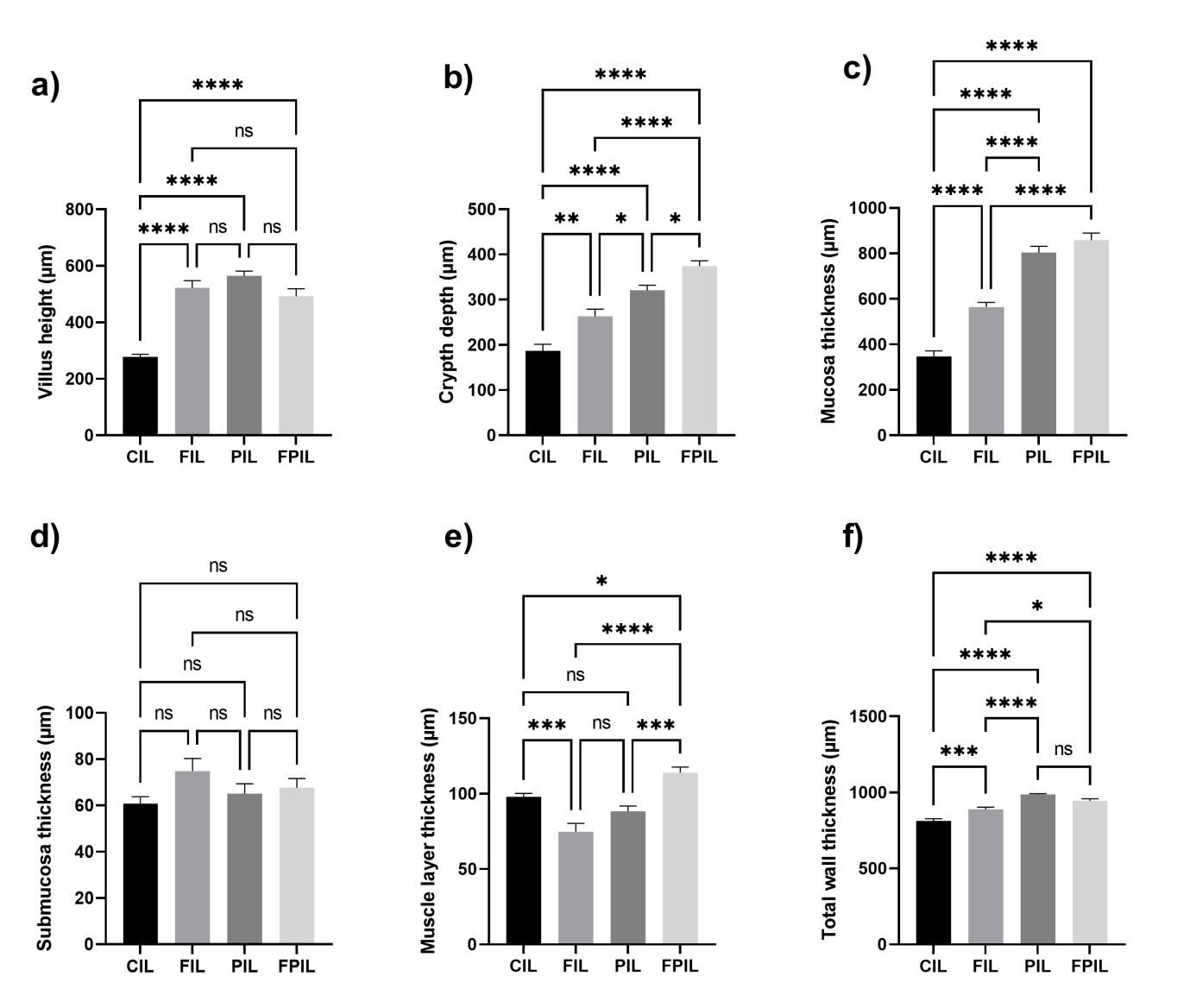


**Fig. S11.** Histomorphometric analysis of intestinal parameters of rat ileum tissues evaluated in all groups. Measurements are shown as (a) villus height (µm), (b) crypth depth (µm), (c) mucosa thickness (µm), (d) submucosa thickness (µm), (e) mucosa layer thickness (µm), and (f) intestinal total wall thickness (µm). CIL (control), FIL (Intermittent fasting), PIL (SCD Probiotics), and the FPIL applications (in which the Intermittent fasting and SCD Probiotics were applied together). P< 0.05 *, P ≤ 0.01 **, P ≤ 0.001 ***, and P ≤ 0.0001 **** (one-way ANOVA test with Tukey's post-hoc test). Results are presented as mean ± SEM (standard error of the mean).

**
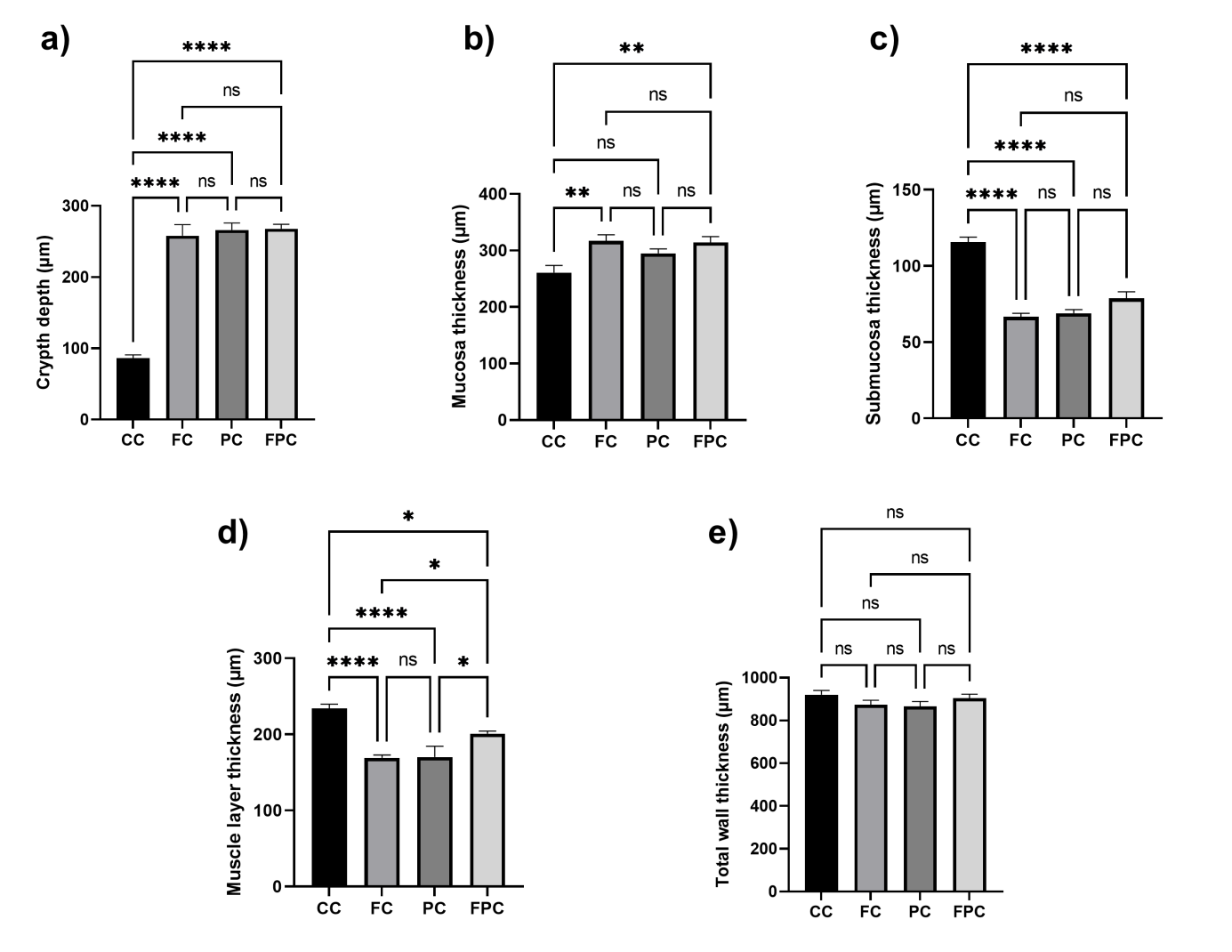
**

**Fig. S12.** Histomorphometric analysis of intestinal parameters of rat colon tissues evaluated in all groups. Measurements are shown as (a) crypth depth (µm), (b) mucosa thickness (µm), (c) submucosa thickness (µm), (d) mucosa layer thickness (µm), and (e) intestinal total wall thickness (µm). CC (control), FC (Intermittent fasting), PC (SDC Probiotics), and the FPC applications (in which the Intermittent fasting and SCD Probiotics were applied together). P< 0.05 *, P ≤ 0.01 ** and P ≤ 0.0001 **** (one-way ANOVA test with Tukey's post-hoc test). Results are presented as mean ± SEM (standard error of the mean).

**
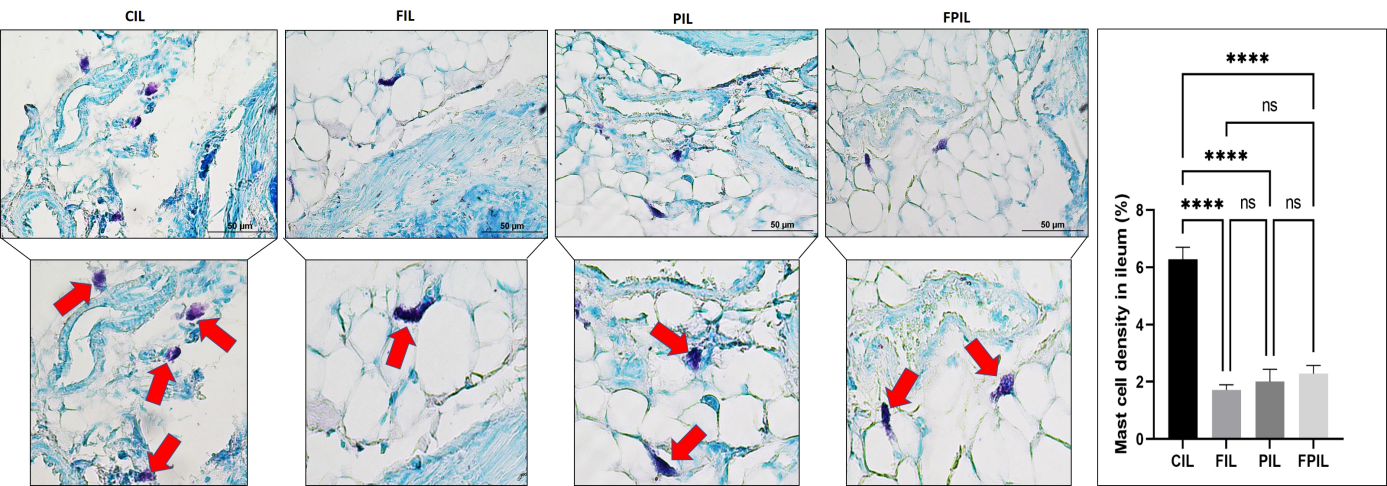
**

**Fig. S13.** Representative toluidine blue staining images of ileum tissue with quantification of mast cell density of area fraction (%) in each group. Red arrows show intestinal serosal mast cells. Graph of TB staining serosal mast cells intensity in the rat ileum as measured in ImageJ FIJI. Values are expressed as mean ±SEM; n = 7 rats in each group. P ≤ 0.0001 **** (nonparametric Mann‐Whitney U test). Scale bar = 50 µm. CIL (control), FIL (Intermittent fasting), PIL (SCD Probiotics), and the FPIL applications (in which the Intermittent fasting and SCD Probiotics were applied together).


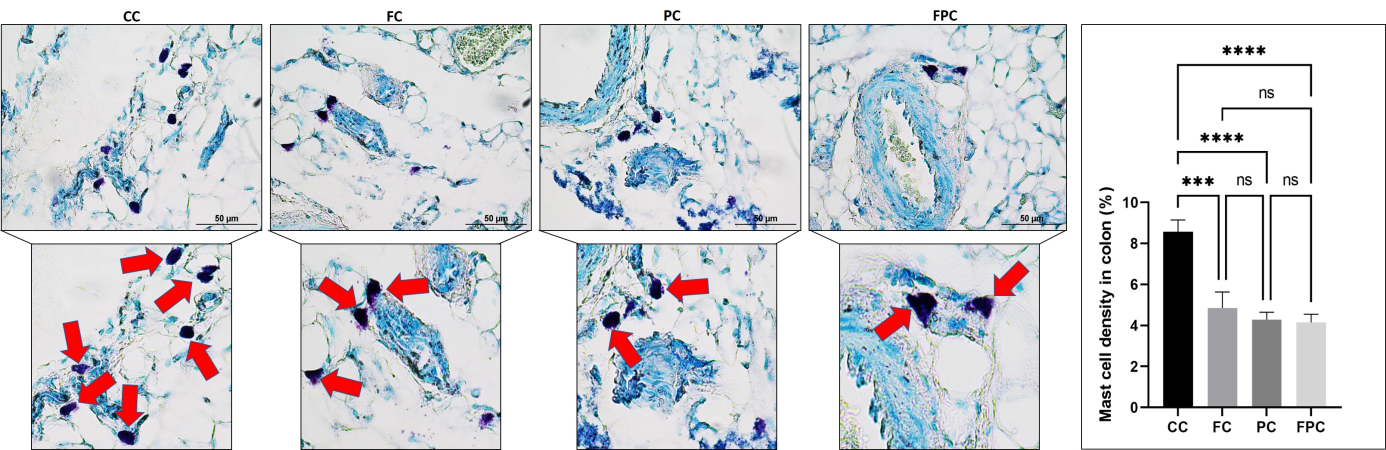


**Fig. S14.** Representative toluidine blue staining images of colon tissue with quantification of mast cell density area fraction (%) in each group. Red arrows show intestinal serosal mast cells. Graph of TB staining serosal mast cells intensity in the rat colon as measured in ImageJ FIJI. Values are expressed as mean ±SEM; n = 7 rats in each group. Values are expressed as mean ±SEM; n = 7 rats in each group. The significance levels were stated as P ≤ 0.001 *** and P ≤ 0.0001 ****. (One-way anova and nonparametric Mann‐Whitney U test).. Scale bar = 50 µm. CC (control), FC (Intermittent fasting), PC (SDC Probiotics), and the FPC applications (in which the Intermittent fasting and SCD Probiotics were applied together).
