# Supplementary material for: Supplementing probiotics during intermittent fasting proves more effective in restoring ileum and colon tissues in aged rats
Source: J Cell Mol Med. 2024 Mar 6;28(6):e18203. doi: 10.1111/jcmm.18203 (PMC10915827; doi:10.1111/jcmm.18203)
Supplement: Supplementary file 2 — Table S1. [file JCMM-28-e18203-s002.docx]

**Supplementing probiotics during intermittent fasting proves more effective in restoring ileum and colon tissues in aged rats**

Hikmet Taner Teker^1^, Taha Ceylani^2,3*^, Seda Keskin^4^, Gizem Samgane^5^, Hüseyin Allahverdi^2^, Eda Acikgoz^4^, Rafig Gurbanov^6,7*^

^1^Department of Molecular Biology, Ankara Medipol University Ankara, Turkey

^2^Department of Molecular Biology and Genetics, Muş Alparslan University Muş, Turkey

^3^Department of Food Quality Control and Analysis, Muş Alparslan University Muş, Turkey

^4^Department of Histology and Embryology, Van Yuzuncu Yil University, Van, Turkey

^5^Institute of Graduate Education, Department Biotechnology, Bilecik Şeyh Edebali University Bilecik, Turkey

^6^Department of Bioengineering, Bilecik Şeyh Edebali University Bilecik, Turkey

^7^Central Research Laboratory, Bilecik Seyh Edebali University Bilecik, Turkey

* Correspondence: [t.ceylani@alparslan.edu.tr](mailto:t.ceylani@alparslan.edu.tr) ORCID ID: 0000-0002-3041-6010 (T.Ceylani), [rafik.kurbanov@gmail.com](mailto:rafik.kurbanov@gmail.com) ORCID ID: 0000-0002-5293-6447 (R. Gurbanov)

**SUPPLEMENTARY TABLES**

**Table S1** LDA confusion matrix for ileum samples in lipid (3000-2700 cm^-1^) spectral region. Control (CIL) and intermittent fasting (FIL), SCD Probiotics (PIL) and the group (FPIL) in which the IF and SCD Probiotics

| **Confusion matrix** | **Actual** | **CIL** | **FIL** | **PIL** | **FPIL** |
| --- | --- | --- | --- | --- | --- |
| Predicted |  | 1 | 2 | 3 | 4 |
| **CIL** | 1 | **12** | 0 | 0 | 0 |
| **FIL** | 2 | 0 | **11** | 0 | 0 |
| **PIL** | 3 | 0 | 0 | **11** | 0 |
| **FPIL** | 4 | 0 | 0 | 0 | **10** |

**Table S2** LDA confusion matrix for ileum samples in lipid (3000-2700 cm^-1^) spectral region. Control (CIL) and intermittent fasting (FIL), SCD Probiotics (PIL) and the group (FPIL) in which the IF and SCD Probiotics

| **Prediction Matrix** | **CIL** | **FIL** | **PIL** | **FPIL** | **Predicted** |
| --- | --- | --- | --- | --- | --- |
|  | 1 | 2 | 3 | 4 | 5 |
| 1 | -477,61 | 2,12 | 39,27 | 6,74 | CIL |
| 2 | -436,15 | -29,97 | 36,60 | 23,86 | CIL |
| 3 | -336,09 | 11,71 | 37,56 | -19,33 | CIL |
| 4 | -628,04 | -5,91 | 37,11 | -41,17 | CIL |
| 5 | -674,90 | -198,72 | 36,79 | -1060,45 | CIL |
| 6 | -395,05 | 12,96 | 38,52 | 3,85 | CIL |
| 7 | -2859,92 | -5266,12 | 35,56 | -34712,50 | CIL |
| 8 | -872,65 | -10,18 | 36,79 | -63,66 | CIL |
| 9 | -3526,63 | -2081,10 | 35,60 | -209,18 | CIL |
| 10 | -1253,42 | -7,59 | 39,24 | -18,53 | CIL |
| 11 | -1042,65 | 12,53 | 37,01 | -26,13 | CIL |
| 12 | -1065,73 | 35,29 | 38,23 | -22,11 | CIL |
| 13 | -6912,87 | 44,59 | -75,36 | -59,52 | FIL |
| 14 | -6729,93 | 42,42 | -52,58 | -98,66 | FIL |
| 15 | -9781,78 | 43,59 | -159,41 | -49,47 | FIL |
| 16 | -9699,74 | 42,34 | -70,97 | -68,65 | FIL |
| 17 | -7802,52 | 44,05 | -88,84 | -37,40 | FIL |
| 18 | -7210,17 | 43,24 | -65,35 | -91,20 | FIL |
| 19 | -11413,03 | 42,01 | -163,10 | -80,18 | FIL |
| 20 | -9218,56 | 41,86 | -146,97 | -96,55 | FIL |
| 21 | -8302,92 | 43,70 | -152,61 | -34,82 | FIL |
| 22 | -6764,70 | 41,65 | -38,38 | -101,18 | FIL |
| 23 | -1068,34 | -234,06 | 7,72 | 38,81 | PIL |
| 24 | -846,54 | -1,92 | -63,06 | 38,62 | PIL |
| 25 | -490,02 | -12,61 | -80,20 | 39,24 | PIL |
| 26 | -1223,42 | -60,78 | 21,81 | 42,62 | PIL |
| 27 | -604,10 | -121,50 | 16,48 | 40,79 | PIL |
| 28 | -686,12 | -89,65 | 25,27 | 41,50 | PIL |
| 29 | -634,23 | -122,01 | 7,25 | 41,58 | PIL |
| 30 | -2857,74 | -817,69 | -91,23 | 38,61 | PIL |
| 31 | -2151,50 | -135,17 | 13,12 | 39,58 | PIL |
| 32 | -1654,50 | -139,00 | 21,43 | 39,28 | PIL |
| 33 | -3346,13 | -199,12 | 0,33 | 38,86 | PIL |
| 34 | -1658,87 | 41,53 | -14,25 | 33,27 | FIL |
| 35 | 41,43 | -31,95 | 9,49 | -133,64 | FPIL |
| 36 | 42,99 | -72,21 | 2,96 | -170,88 | FPIL |
| 37 | 41,63 | -162,00 | -12,90 | -200,88 | FPIL |
| 38 | 41,35 | -1862,66 | -146,50 | -215,27 | FPIL |
| 39 | 41,69 | -91,70 | -28,95 | -187,23 | FPIL |
| 40 | 42,12 | -269,14 | -1,76 | -195,21 | FPIL |
| 41 | 44,10 | -46,40 | -5,64 | -185,12 | FPIL |
| 42 | 42,07 | -46,09 | 2,79 | -171,20 | FPIL |
| 43 | 42,02 | -138,88 | -38,28 | -208,14 | FPIL |
| 44 | 41,38 | -187,54 | -12,75 | -512,88 | FPIL |

**Table S3** LDA confusion matrix for ileum samples in protein (1700-1500 cm^-1^) spectral region. Control (CIL) and intermittent fasting (FIL), SCD Probiotics (PIL) and the group (FPIL) in which the IF and SCD Probiotics

| **Confusion matrix** | **Actual** | **CIL** | **FIL** | **PIL** | **FPIL** |
| --- | --- | --- | --- | --- | --- |
| Predicted |  | 1 | 2 | 3 | 4 |
| **CIL** | 1 | **12** | 0 | 0 | 0 |
| **FIL** | 2 | 0 | **11** | 0 | 0 |
| **PIL** | 3 | 0 | 0 | **11** | 0 |
| **FPIL** | 4 | 0 | 0 | 0 | **10** |

**Table S4** LDA confusion matrix for ileum samples in protein (1700-1500 cm^-1^) spectral region. Control (CIL) and intermittent fasting (FIL), SCD Probiotics (PIL) and the group (FPIL) in which the IF and SCD Probiotics

| **Prediction Matrix** | **CIL** | **FIL** | **PIL** | **FPIL** | **Predicted** |
| --- | --- | --- | --- | --- | --- |
|  | 1 | 2 | 3 | 4 | 5 |
| 1 | 29,34 | 13,25 | -898,60 | -1291,34 | CIL |
| 2 | 27,20 | 23,63 | -482,69 | -1040,55 | CIL |
| 3 | 29,35 | -9,95 | -855,14 | -1424,01 | CIL |
| 4 | 27,10 | -2,92 | -694,28 | -1421,69 | CIL |
| 5 | 26,73 | 3,00 | -331,15 | -2239,77 | CIL |
| 6 | 26,24 | 12,65 | -628,81 | -2610,84 | CIL |
| 7 | 26,24 | -175,12 | -86,72 | -1565,74 | CIL |
| 8 | 27,54 | -8,53 | -561,94 | -1861,81 | CIL |
| 9 | 27,01 | -25,49 | -595,37 | -917,73 | CIL |
| 10 | 29,47 | 9,97 | -739,87 | -1794,25 | CIL |
| 11 | 28,60 | -2,75 | -1021,14 | -2056,18 | CIL |
| 12 | 30,24 | 8,65 | -808,38 | -1572,93 | CIL |
| 13 | -9,73 | 30,96 | -172,62 | -1771,48 | FIL |
| 14 | 21,81 | 28,40 | -335,25 | -1991,62 | FIL |
| 15 | 16,65 | 31,03 | -62,27 | -1655,10 | FIL |
| 16 | -79,25 | 29,11 | -84,56 | -2143,20 | FIL |
| 17 | 14,47 | 28,40 | -166,34 | -2522,72 | FIL |
| 18 | -14,32 | 28,91 | -169,88 | -2236,01 | FIL |
| 19 | 1,78 | 30,77 | -64,18 | -1585,22 | FIL |
| 20 | 23,47 | 28,48 | -93,23 | -1610,62 | FIL |
| 21 | 18,02 | 31,91 | -64,09 | -1763,95 | FIL |
| 22 | -97,34 | 30,24 | -147,32 | -2123,02 | FIL |
| 23 | -223,53 | -20,93 | 28,55 | -2697,87 | PIL |
| 24 | -82,13 | 16,23 | 27,77 | -2114,60 | PIL |
| 25 | 1,81 | -8,02 | 27,86 | -2500,59 | PIL |
| 26 | -19,42 | -2,30 | 28,92 | -2925,45 | PIL |
| 27 | -100,83 | 3,44 | 28,30 | -2836,37 | PIL |
| 28 | -187,17 | -11,19 | 29,08 | -3482,49 | PIL |
| 29 | -5,89 | 4,20 | 28,84 | -2882,34 | PIL |
| 30 | -14,62 | -3,86 | 28,46 | -2448,35 | PIL |
| 31 | -108,05 | 12,56 | 29,55 | -3755,89 | PIL |
| 32 | -10,65 | 23,95 | 29,50 | -3270,09 | PIL |
| 33 | -77,34 | 15,58 | 30,38 | -2894,34 | PIL |
| 34 | 8,65 | 28,46 | 22,83 | -2951,02 | FIL |
| 35 | -60,90 | -224,48 | -1788,27 | 30,51 | FPIL |
| 36 | -41,52 | -208,80 | -2228,88 | 29,65 | FPIL |
| 37 | -187,90 | -254,37 | -1544,36 | 31,93 | FPIL |
| 38 | -646,68 | -284,20 | -1134,79 | 29,08 | FPIL |
| 39 | -224,89 | -269,04 | -1539,04 | 30,66 | FPIL |
| 40 | -101,66 | -266,18 | -1741,24 | 29,08 | FPIL |
| 41 | -43,88 | -325,48 | -1521,21 | 29,23 | FPIL |
| 42 | -104,85 | -267,93 | -1677,65 | 29,82 | FPIL |
| 43 | -142,75 | -261,42 | -1981,08 | 30,43 | FPIL |
| 44 | -45,37 | -240,96 | -1948,64 | 29,34 | FPIL |

**Table S5** LDA confusion matrix for ileum samples in spectral region in nucleic acids and polysaccharides (1200-650 cm^-1^). Control (CIL) and intermittent fasting (FIL), SCD Probiotics (PIL) and the group (FPIL) in which the IF and SCD Probiotics

| **Confusion matrix** | **Actual** | **CIL** | **FIL** | **PIL** | **FPIL** |
| --- | --- | --- | --- | --- | --- |
| Predicted |  | 1 | 2 | 3 | 4 |
| **CIL** | 1 | **12** | 0 | 0 | 0 |
| **FIL** | 2 | 0 | **11** | 0 | 0 |
| **PIL** | 3 | 0 | 0 | **11** | 0 |
| **FPIL** | 4 | 0 | 0 | 0 | **10** |

**Table S6** LDA confusion matrix for ileum samples in spectral region in nucleic acids and polysaccharides (1200-650 cm^-1^). Control (CIL) and intermittent fasting (FIL), SCD Probiotics (PIL) and the group (FPIL) in which the IF and SCD Probiotics

| **Prediction Matrix** | **CIL** | **FIL** | **PIL** | **FPIL** | **Predicted** |
| --- | --- | --- | --- | --- | --- |
|  | 1 | 2 | 3 | 4 | 5 |
| 1 | 25,74 | -224,88 | -11760,69 | -70,50 | CIL |
| 2 | 24,58 | -119,41 | -9269,40 | -137,61 | CIL |
| 3 | 26,47 | -399,85 | -11398,90 | -65,36 | CIL |
| 4 | 25,54 | -447,25 | -14460,58 | -84,33 | CIL |
| 5 | 24,64 | -1929,28 | -3629,94 | -370,93 | CIL |
| 6 | 26,18 | -107,90 | -9699,63 | -160,19 | CIL |
| 7 | 23,60 | -22405,53 | -85335,88 | -7815,87 | CIL |
| 8 | 24,16 | -166,20 | -10357,09 | -195,41 | CIL |
| 9 | 24,36 | -1517,96 | -9895,78 | -247,85 | CIL |
| 10 | 28,11 | -321,26 | -10545,24 | -144,44 | CIL |
| 11 | 25,93 | -202,19 | -13952,91 | -164,95 | CIL |
| 12 | 25,43 | -353,52 | -12843,99 | -208,68 | CIL |
| 13 | 5,58 | 31,83 | -6027,46 | -543,19 | FIL |
| 14 | 2,07 | 31,20 | -6108,83 | -535,44 | FIL |
| 15 | -28,65 | 30,48 | -4634,90 | -755,17 | FIL |
| 16 | 0,94 | 31,59 | -4051,94 | -532,72 | FIL |
| 17 | 3,14 | 31,93 | -3976,44 | -571,46 | FIL |
| 18 | -10,39 | 31,06 | -5153,83 | -650,34 | FIL |
| 19 | -47,92 | 32,70 | -3102,47 | -902,61 | FIL |
| 20 | -66,41 | 32,02 | -3616,38 | -934,49 | FIL |
| 21 | -46,66 | 31,25 | -2696,61 | -934,68 | FIL |
| 22 | -4,93 | 32,37 | -3226,70 | -717,57 | FIL |
| 23 | -61,80 | -164,06 | 32,17 | -1367,07 | PIL |
| 24 | -90,11 | -81,86 | 31,33 | -1606,38 | PIL |
| 25 | -70,05 | -153,64 | 31,45 | -1578,73 | PIL |
| 26 | -54,94 | -98,21 | 33,66 | -1321,17 | PIL |
| 27 | -55,79 | -85,25 | 34,66 | -1336,50 | PIL |
| 28 | -53,71 | -128,94 | 31,37 | -1146,59 | PIL |
| 29 | -57,31 | -111,70 | 33,24 | -1401,23 | PIL |
| 30 | -50,33 | -111,76 | 31,43 | -1364,55 | PIL |
| 31 | -48,04 | -42,95 | 31,54 | -1105,16 | PIL |
| 32 | -152,84 | -0,60 | 32,13 | -2240,23 | PIL |
| 33 | -298,13 | 21,33 | 31,10 | -3560,16 | PIL |
| 34 | -468,28 | 30,17 | -90,15 | -5300,62 | FIL |
| 35 | -18,87 | -828,95 | -12228,20 | 31,41 | FPIL |
| 36 | -17,38 | -1139,91 | -16106,37 | 31,69 | FPIL |
| 37 | 6,88 | -1477,39 | -11759,83 | 30,44 | FPIL |
| 38 | 1,46 | -2191,20 | -14002,49 | 30,04 | FPIL |
| 39 | -4,59 | -1406,80 | -10355,61 | 30,39 | FPIL |
| 40 | -35,43 | -1196,83 | -11512,30 | 30,35 | FPIL |
| 41 | -42,22 | -586,61 | -12149,82 | 29,89 | FPIL |
| 42 | -37,26 | -754,94 | -11396,13 | 31,88 | FPIL |
| 43 | -43,47 | -867,76 | -17281,23 | 30,77 | FPIL |
| 44 | -35,26 | -1002,94 | -16968,54 | 29,82 | FPIL |

**Table S7** LDA confusion matrix for ileum samples in the full (4000-650 cm^-1^) spectral region. Control (CIL) and intermittent fasting (FIL), SCD Probiotics (PIL) and the group (FPIL) in which the IF and SCD Probiotics

| **Confusion matrix** | **Actual** | **CC** | **FC** | **PC** | **FPC** |
| --- | --- | --- | --- | --- | --- |
| Predicted |  | 1 | 2 | 3 | 4 |
| **CC** | 1 | **12** | 0 | 0 | 0 |
| **FC** | 2 | 0 | **10** | 1 | 0 |
| **PC** | 3 | 0 | 0 | **11** | 0 |
| **FPC** | 4 | 0 | 0 | 0 | **10** |

**Table S8** LDA confusion matrix for ileum samples in the full (4000-650 cm^-1^) spectral region. Control (CIL) and intermittent fasting (FIL), SCD Probiotics (PIL) and the group (FPIL) in which the IF and SCD Probiotics

| **Prediction Matrix** | **CC** | **FC** | **PC** | **FPC** | **Predicted** |
| --- | --- | --- | --- | --- | --- |
|  | 1 | 2 | 3 | 4 | 5 |
| 1 | 15,57 | -5869,93 | -975,08 | -274,23 | CC |
| 2 | 13,39 | -6516,19 | -913,50 | -363,92 | CC |
| 3 | 14,27 | -7330,13 | -1359,88 | -1568,27 | CC |
| 4 | 13,88 | -13224,19 | -2089,97 | -3287,85 | CC |
| 5 | 14,46 | -36683,30 | -6329,20 | -13329,94 | CC |
| 6 | 13,36 | -66002,61 | -13894,24 | -33748,53 | CC |
| 7 | 15,01 | -2975,18 | -433,86 | -436,56 | CC |
| 8 | 14,78 | -7464,15 | -1151,69 | -126,14 | CC |
| 9 | 15,34 | -8016,48 | -1127,59 | -197,68 | CC |
| 10 | 15,55 | -5937,26 | -979,99 | -210,52 | CC |
| 11 | 15,97 | -2971,27 | -468,92 | -164,01 | CC |
| 12 | 15,64 | -4194,02 | -575,72 | -153,21 | CC |
| 13 | -12,06 | 22,96 | -2,24 | -759,70 | FC |
| 14 | -35,33 | 21,36 | 17,04 | -1182,76 | FC |
| 15 | -27,13 | 22,77 | 10,25 | -784,87 | FC |
| 16 | -8,42 | 22,15 | 16,92 | -819,85 | FC |
| 17 | -59,52 | 22,03 | -35,38 | -1009,09 | FC |
| 18 | -54,53 | 21,41 | -96,71 | -890,28 | FC |
| 19 | -20,54 | 24,05 | -0,38 | -882,12 | FC |
| 20 | -16,63 | 22,29 | 16,70 | -793,27 | FC |
| 21 | -8,75 | 22,03 | -17,93 | -793,74 | FC |
| 22 | -9,24 | 21,43 | 16,94 | -707,80 | FC |
| 23 | -31,54 | -192,99 | 20,94 | -698,36 | PC |
| 24 | -38,05 | -9,01 | 20,92 | -1003,81 | PC |
| 25 | -18,88 | 22,89 | 22,01 | -961,91 | FC |
| 26 | -14,10 | -132,49 | 21,30 | -730,42 | PC |
| 27 | -9,38 | -12,52 | 24,10 | -626,92 | PC |
| 28 | -1,82 | -24,62 | 21,98 | -624,00 | PC |
| 29 | -10,86 | -10,56 | 23,20 | -714,67 | PC |
| 30 | -6,90 | -6,93 | 22,22 | -786,41 | PC |
| 31 | -6,80 | 4,15 | 22,19 | -791,00 | PC |
| 32 | -9,34 | 10,07 | 22,64 | -579,01 | PC |
| 33 | -7,40 | 18,75 | 22,94 | -666,23 | PC |
| 34 | -4,10 | 1,81 | 23,07 | -622,41 | PC |
| 35 | -55,66 | -8511,11 | -1331,07 | 22,54 | FPC |
| 36 | -60,69 | -9837,80 | -1157,92 | 22,93 | FPC |
| 37 | -22,60 | -10954,59 | -1510,02 | 22,25 | FPC |
| 38 | -62,08 | -9977,31 | -1118,95 | 22,34 | FPC |
| 39 | -52,87 | -8875,52 | -1503,33 | 22,11 | FPC |
| 40 | -57,89 | -7484,22 | -1256,23 | 22,75 | FPC |
| 41 | -26,30 | -8268,42 | -1337,80 | 23,83 | FPC |
| 42 | -44,84 | -8811,27 | -1058,39 | 22,39 | FPC |
| 43 | -22,10 | -8302,33 | -1383,60 | 24,45 | FPC |
| 44 | -17,21 | -7691,24 | -1336,25 | 24,19 | FPC |

**Table S9** LDA confusion matrix for colon samples in lipid (3000-2700 cm^-1^) spectral region. Control (CC) and intermittent fasting (FC), SCD Probiotics (PC) and the group (FPC) in which the IF and SCD Probiotics

| **Confusion matrix** | **Actual** | **CC** | **FC** | **PC** | **FPC** |
| --- | --- | --- | --- | --- | --- |
| Predicted |  | 1 | 2 | 3 | 4 |
| **CC** | 1 | **12** | 0 | 0 | 0 |
| **FC** | 2 | 0 | **9** | 1 | 0 |
| **PC** | 3 | 0 | 1 | **11** | 0 |
| **FPC** | 4 | 0 | 0 | 0 | **10** |

**Table S10** LDA confusion matrix for colon samples in lipid (3000-2700 cm^-1^) spectral region. Control (CC) and intermittent fasting (FC), SCD Probiotics (PC) and the group (FPC) in which the IF and SCD Probiotics

| **Prediction Matrix** | **CC** | **FC** | **PC** | **FPC** | **Predicted** |
| --- | --- | --- | --- | --- | --- |
|  | 1 | 2 | 3 | 4 | 5 |
| 1 | 35,35 | -448,83 | -232,73 | 4,87 | CC |
| 2 | 33,23 | -1159,59 | -266,35 | -183,32 | CC |
| 3 | 33,71 | -603,60 | -332,02 | -60,70 | CC |
| 4 | 33,40 | -476,08 | -124,19 | -167,16 | CC |
| 5 | 36,63 | -147,46 | 7,72 | -284,16 | CC |
| 6 | 33,27 | 6,59 | -60,45 | -3300,98 | CC |
| 7 | 36,50 | -124,06 | -9,75 | -175,12 | CC |
| 8 | 35,29 | -486,44 | -170,57 | -66,75 | CC |
| 9 | 35,03 | -233,05 | -57,01 | -52,86 | CC |
| 10 | 35,63 | -322,64 | -136,07 | -25,48 | CC |
| 11 | 33,32 | -65,48 | -8,35 | -80,70 | CC |
| 12 | 34,62 | -134,32 | 2,86 | -149,99 | CC |
| 13 | 35,83 | 38,67 | 19,08 | -1168,70 | FC |
| 14 | 5,89 | 38,21 | -2,25 | -108,23 | FC |
| 15 | 30,22 | 38,45 | 29,98 | -756,02 | FC |
| 16 | 31,47 | 41,02 | 17,85 | -684,99 | FC |
| 17 | 3,27 | 38,73 | 32,15 | -487,03 | FC |
| 18 | 8,72 | 38,36 | 27,04 | -418,74 | FC |
| 19 | 28,24 | 39,24 | 35,63 | -476,60 | FC |
| 20 | 24,75 | 38,27 | 40,00 | -276,28 | PC |
| 21 | 25,36 | 39,01 | 27,93 | -557,85 | FC |
| 22 | 28,15 | 40,20 | 34,57 | -433,04 | FC |
| 23 | 11,72 | 38,73 | 37,78 | -299,74 | FC |
| 24 | -20,27 | -285,80 | 36,68 | -145,80 | PC |
| 25 | 7,82 | 8,41 | 36,80 | 0,88 | PC |
| 26 | 23,99 | -0,88 | 39,08 | -157,26 | PC |
| 27 | 23,25 | 19,69 | 40,65 | -264,55 | PC |
| 28 | 29,77 | 11,32 | 40,32 | -180,09 | PC |
| 29 | 30,29 | 36,61 | 37,81 | -478,93 | PC |
| 30 | 32,22 | 21,91 | 39,47 | -292,85 | PC |
| 31 | 30,19 | 14,71 | 37,90 | -221,58 | PC |
| 32 | 22,90 | 8,50 | 38,12 | -308,02 | PC |
| 33 | 21,40 | 33,48 | 38,31 | -239,07 | PC |
| 34 | 25,19 | 33,89 | 38,31 | -231,89 | PC |
| 35 | 20,27 | -330,19 | -85,52 | 40,71 | FPC |
| 36 | -1,01 | -826,58 | -100,79 | 40,08 | FPC |
| 37 | 17,47 | -467,36 | -68,71 | 39,95 | FPC |
| 38 | 1,97 | -634,59 | -116,24 | 40,74 | FPC |
| 39 | 22,93 | -520,00 | -153,75 | 41,04 | FPC |
| 40 | 20,82 | -483,74 | -170,10 | 39,66 | FPC |
| 41 | 19,03 | -382,06 | -30,74 | 39,90 | FPC |
| 42 | 6,40 | -788,86 | -138,21 | 41,35 | FPC |
| 43 | -1,53 | -596,65 | -120,01 | 39,93 | FPC |
| 44 | 20,12 | -490,63 | -119,04 | 40,95 | FPC |

**Table S11** LDA confusion matrix for colon samples in protein (1700-1500 cm^-1^) spectral region. Control (CC) and intermittent fasting (FC), SCD Probiotics (PC) and the group (FPC) in which the IF and SCD Probiotics

| **Confusion matrix** | **Actual** | **CC** | **FC** | **PC** | **FPC** |
| --- | --- | --- | --- | --- | --- |
| Predicted |  | 1 | 2 | 3 | 4 |
| **CC** | 1 | **12** | 0 | 0 | 0 |
| **FC** | 2 | 0 | **8** | 1 | 0 |
| **PC** | 3 | 0 | 2 | **11** | 0 |
| **FPC** | 4 | 0 | 0 | 0 | **10** |

**Table S12** LDA confusion matrix for colon samples in protein (1700-1500 cm^-1^) spectral region. Control (CC) and intermittent fasting (FC), SCD Probiotics (PC) and the group (FPC) in which the IF and SCD Probiotics

| **Prediction Matrix** | **CC** | **FC** | **PC** | **FPC** | **Predicted** |
| --- | --- | --- | --- | --- | --- |
|  | 1 | 2 | 3 | 4 | 5 |
| 1 | 27,16 | -65,14 | -389,12 | -55,17 | CC |
| 2 | 25,42 | -60,43 | -268,08 | -87,16 | CC |
| 3 | 23,49 | -31,31 | -156,11 | -173,58 | CC |
| 4 | 23,96 | -163,08 | -221,87 | -88,38 | CC |
| 5 | 25,74 | -91,64 | -318,23 | -104,23 | CC |
| 6 | 25,15 | -101,38 | -597,35 | -104,37 | CC |
| 7 | 24,70 | -10,57 | -64,84 | -36,22 | CC |
| 8 | 25,35 | -94,43 | -525,27 | -67,89 | CC |
| 9 | 26,01 | -40,57 | -226,76 | -52,30 | CC |
| 10 | 23,72 | -99,48 | -388,14 | -103,02 | CC |
| 11 | 24,03 | -1,42 | -124,03 | -64,39 | CC |
| 12 | 25,27 | -21,29 | -43,00 | -33,17 | CC |
| 13 | -20,08 | 26,81 | 27,41 | -47,64 | PC |
| 14 | -43,18 | 26,84 | -7,46 | 4,42 | FC |
| 15 | -44,54 | 27,17 | 20,86 | -34,20 | FC |
| 16 | -16,68 | 26,90 | 27,53 | -38,80 | PC |
| 17 | -113,27 | 27,23 | 16,58 | -51,16 | FC |
| 18 | -61,08 | 26,71 | -70,29 | -132,09 | FC |
| 19 | -40,19 | 29,04 | 24,64 | -72,08 | FC |
| 20 | -29,65 | 27,84 | 25,71 | -46,78 | FC |
| 21 | -16,89 | 28,22 | 20,71 | -126,08 | FC |
| 22 | -10,68 | 29,14 | 22,02 | -99,19 | FC |
| 23 | -70,30 | 28,18 | 27,10 | -40,25 | FC |
| 24 | -44,94 | -17,80 | 27,16 | -41,32 | PC |
| 25 | -42,05 | 11,21 | 28,13 | -17,22 | PC |
| 26 | -25,88 | 25,70 | 28,44 | -26,90 | PC |
| 27 | -14,46 | 26,53 | 28,68 | -53,37 | PC |
| 28 | -5,91 | 24,54 | 27,37 | -54,56 | PC |
| 29 | -27,04 | 28,01 | 31,07 | -28,47 | PC |
| 30 | -18,88 | 15,45 | 28,42 | -55,78 | PC |
| 31 | -13,62 | 23,27 | 29,99 | -32,68 | PC |
| 32 | -33,06 | 15,27 | 29,09 | -78,48 | PC |
| 33 | -10,44 | 16,94 | 29,43 | -87,61 | PC |
| 34 | -10,77 | 23,65 | 28,57 | -67,09 | PC |
| 35 | -54,03 | 7,95 | -398,69 | 27,64 | FPC |
| 36 | -49,98 | 6,17 | -207,02 | 27,25 | FPC |
| 37 | -32,55 | -16,99 | -451,81 | 26,77 | FPC |
| 38 | -71,89 | -20,23 | -275,87 | 28,38 | FPC |
| 39 | -20,75 | 11,56 | -409,49 | 26,63 | FPC |
| 40 | -37,06 | 4,78 | -366,60 | 27,50 | FPC |
| 41 | -21,54 | -17,92 | -362,99 | 27,57 | FPC |
| 42 | -49,88 | -8,07 | -215,20 | 26,51 | FPC |
| 43 | -7,20 | -37,36 | -258,79 | 26,49 | FPC |
| 44 | -13,05 | 3,90 | -292,50 | 26,89 | FPC |

**Table S13** LDA confusion matrix for colon samples in spectral region in nucleic acids and polysaccharides (1200-650 cm^-1^). Control (CC) and intermittent fasting (FC), SCD Probiotics (PC) and the group (FPC) in which the IF and SCD Probiotics

| **Confusion matrix** | **Actual** | **CC** | **FC** | **PC** | **FPC** |
| --- | --- | --- | --- | --- | --- |
| Predicted |  | 1 | 2 | 3 | 4 |
| **CC** | 1 | **12** | 0 | 0 | 0 |
| **FC** | 2 | 0 | **10** | 1 | 0 |
| **PC** | 3 | 0 | 0 | **11** | 0 |
| **FPC** | 4 | 0 | 0 | 0 | **10** |

**Table S14** LDA confusion matrix for colon samples in spectral region in nucleic acids and polysaccharides (1200-650 cm^-1^). Control (CC) and intermittent fasting (FC), SCD Probiotics (PC) and the group (FPC) in which the IF and SCD Probiotics

| **Prediction Matrix** | **CC** | **FC** | **PC** | **FPC** | **Predicted** |
| --- | --- | --- | --- | --- | --- |
|  | 1 | 2 | 3 | 4 | 5 |
| 1 | 24,72 | -1728,40 | -675,56 | -146,33 | CC |
| 2 | 22,85 | -386,20 | -836,00 | -304,68 | CC |
| 3 | 23,03 | -164,91 | -719,17 | -466,26 | CC |
| 4 | 22,15 | -670,52 | -805,17 | -607,65 | CC |
| 5 | 22,65 | -351,99 | -1220,47 | -791,34 | CC |
| 6 | 23,02 | -382,27 | -1412,54 | -1071,97 | CC |
| 7 | 24,11 | -229,74 | -243,99 | -243,12 | CC |
| 8 | 23,58 | -2880,93 | -709,43 | -32,22 | CC |
| 9 | 25,01 | -2009,59 | -492,20 | -15,83 | CC |
| 10 | 24,73 | -2337,82 | -623,41 | -60,88 | CC |
| 11 | 25,21 | -1165,05 | -220,59 | -57,12 | CC |
| 12 | 25,14 | -1452,36 | -208,20 | -35,30 | CC |
| 13 | -13,36 | 30,35 | 26,82 | -502,35 | FC |
| 14 | -10,97 | 28,70 | 24,26 | -525,05 | FC |
| 15 | -28,79 | 29,35 | 23,29 | -566,94 | FC |
| 16 | -5,12 | 28,96 | 28,09 | -611,22 | FC |
| 17 | -53,13 | 29,09 | -4,42 | -762,29 | FC |
| 18 | -49,97 | 28,62 | -4,71 | -561,11 | FC |
| 19 | -18,32 | 31,28 | 17,93 | -644,70 | FC |
| 20 | -11,01 | 29,41 | 26,99 | -567,24 | FC |
| 21 | -16,13 | 29,85 | 28,64 | -559,96 | FC |
| 22 | -13,77 | 29,25 | 27,73 | -508,58 | FC |
| 23 | -16,56 | -19,52 | 27,13 | -510,59 | PC |
| 24 | -1,48 | -18,88 | 27,05 | -343,66 | PC |
| 25 | -2,02 | 27,71 | 27,18 | -517,00 | FC |
| 26 | -4,75 | -18,90 | 26,82 | -490,64 | PC |
| 27 | -13,51 | -197,27 | 26,60 | -793,69 | PC |
| 28 | -1,24 | 18,95 | 29,77 | -549,09 | PC |
| 29 | -10,68 | 25,77 | 28,34 | -603,44 | PC |
| 30 | -5,14 | 18,23 | 29,63 | -600,44 | PC |
| 31 | -1,44 | 19,34 | 29,08 | -545,22 | PC |
| 32 | -15,91 | -130,85 | 28,41 | -544,56 | PC |
| 33 | -8,12 | 9,95 | 29,05 | -597,14 | PC |
| 34 | -8,17 | -79,80 | 28,84 | -550,28 | PC |
| 35 | -20,63 | -1206,79 | -297,47 | 28,02 | FPC |
| 36 | -16,43 | -1194,98 | -382,41 | 27,80 | FPC |
| 37 | 12,32 | -2263,28 | -370,82 | 27,12 | FPC |
| 38 | -13,82 | -1808,85 | -367,07 | 27,50 | FPC |
| 39 | -23,52 | -1470,43 | -405,80 | 28,76 | FPC |
| 40 | -31,60 | -1389,28 | -387,82 | 28,70 | FPC |
| 41 | 11,95 | -1487,25 | -241,24 | 27,75 | FPC |
| 42 | -8,78 | -1146,20 | -349,25 | 28,11 | FPC |
| 43 | 3,08 | -1352,93 | -320,03 | 27,30 | FPC |
| 44 | 1,37 | -1791,06 | -351,07 | 28,64 | FPC |

**Table S15** LDA confusion matrix for colon samples in the full (4000-650 cm^-1^) spectral region. Control (CC) and intermittent fasting (FC), SCD Probiotics (PC) and the group (FPC) in which the IF and SCD Probiotics

| **Confusion matrix** | **Actual** | **CC** | **FC** | **PC** | **FPC** |
| --- | --- | --- | --- | --- | --- |
| Predicted |  | 1 | 2 | 3 | 4 |
| **CC** | 1 | **12** | 0 | 0 | 0 |
| **FC** | 2 | 0 | **10** | 1 | 0 |
| **PC** | 3 | 0 | 0 | **11** | 0 |
| **FPC** | 4 | 0 | 0 | 0 | **10** |

**Table S16** LDA confusion matrix for colon samples in the full (4000-650 cm^-1^) spectral region. Control (CC) and intermittent fasting (FC), SCD Probiotics (PC) and the group (FPC) in which the IF and SCD Probiotics

| **Prediction Matrix** | **CC** | **FC** | **PC** | **FPC** | **Predicted** |
| --- | --- | --- | --- | --- | --- |
|  | 1 | 2 | 3 | 4 | 5 |
| 1 | 15,57 | -5869,93 | -975,08 | -274,23 | CC |
| 2 | 13,39 | -6516,19 | -913,50 | -363,92 | CC |
| 3 | 14,27 | -7330,13 | -1359,88 | -1568,27 | CC |
| 4 | 13,88 | -13224,19 | -2089,97 | -3287,85 | CC |
| 5 | 14,46 | -36683,30 | -6329,20 | -13329,94 | CC |
| 6 | 13,36 | -66002,61 | -13894,24 | -33748,53 | CC |
| 7 | 15,01 | -2975,18 | -433,86 | -436,56 | CC |
| 8 | 14,78 | -7464,15 | -1151,69 | -126,14 | CC |
| 9 | 15,34 | -8016,48 | -1127,59 | -197,68 | CC |
| 10 | 15,55 | -5937,26 | -979,99 | -210,52 | CC |
| 11 | 15,97 | -2971,27 | -468,92 | -164,01 | CC |
| 12 | 15,64 | -4194,02 | -575,72 | -153,21 | CC |
| 13 | -12,06 | 22,96 | -2,24 | -759,70 | FC |
| 14 | -35,33 | 21,36 | 17,04 | -1182,76 | FC |
| 15 | -27,13 | 22,77 | 10,25 | -784,87 | FC |
| 16 | -8,42 | 22,15 | 16,92 | -819,85 | FC |
| 17 | -59,52 | 22,03 | -35,38 | -1009,09 | FC |
| 18 | -54,53 | 21,41 | -96,71 | -890,28 | FC |
| 19 | -20,54 | 24,05 | -0,38 | -882,12 | FC |
| 20 | -16,63 | 22,29 | 16,70 | -793,27 | FC |
| 21 | -8,75 | 22,03 | -17,93 | -793,74 | FC |
| 22 | -9,24 | 21,43 | 16,94 | -707,80 | FC |
| 23 | -31,54 | -192,99 | 20,94 | -698,36 | PC |
| 24 | -38,05 | -9,01 | 20,92 | -1003,81 | PC |
| 25 | -18,88 | 22,89 | 22,01 | -961,91 | FC |
| 26 | -14,10 | -132,49 | 21,30 | -730,42 | PC |
| 27 | -9,38 | -12,52 | 24,10 | -626,92 | PC |
| 28 | -1,82 | -24,62 | 21,98 | -624,00 | PC |
| 29 | -10,86 | -10,56 | 23,20 | -714,67 | PC |
| 30 | -6,90 | -6,93 | 22,22 | -786,41 | PC |
| 31 | -6,80 | 4,15 | 22,19 | -791,00 | PC |
| 32 | -9,34 | 10,07 | 22,64 | -579,01 | PC |
| 33 | -7,40 | 18,75 | 22,94 | -666,23 | PC |
| 34 | -4,10 | 1,81 | 23,07 | -622,41 | PC |
| 35 | -55,66 | -8511,11 | -1331,07 | 22,54 | FPC |
| 36 | -60,69 | -9837,80 | -1157,92 | 22,93 | FPC |
| 37 | -22,60 | -10954,59 | -1510,02 | 22,25 | FPC |
| 38 | -62,08 | -9977,31 | -1118,95 | 22,34 | FPC |
| 39 | -52,87 | -8875,52 | -1503,33 | 22,11 | FPC |
| 40 | -57,89 | -7484,22 | -1256,23 | 22,75 | FPC |
| 41 | -26,30 | -8268,42 | -1337,80 | 23,83 | FPC |
| 42 | -44,84 | -8811,27 | -1058,39 | 22,39 | FPC |
| 43 | -22,10 | -8302,33 | -1383,60 | 24,45 | FPC |
| 44 | -17,21 | -7691,24 | -1336,25 | 24,19 | FPC |
